# One-carbon-mediated purine synthesis underlies temozolomide resistance in glioblastoma

**DOI:** 10.1038/s41419-024-07170-y

**Published:** 2024-10-25

**Authors:** Kimia Ghannad-Zadeh, Alyona Ivanova, Megan Wu, Taylor M. Wilson, Alyssa Lau, Robert Flick, David G. Munoz, Sunit Das

**Affiliations:** 1 The Arthur and Sonia Labatt Brain Tumor Research Center, Hospital for Sick Children, Toronto, ON Canada; 2 Institute of Medical Science, Temerty Faculty of Medicine, University of Toronto, Toronto, ON Canada; 3 Department of Chemical Engineering and Applied Chemistry, University of Toronto, Toronto, ON Canada; 4 Department of Laboratory Medicine and Pathobiology, University of Toronto, Toronto, ON Canada; 5 Neurosurgery Research Department, St. Michael’s Hospital, Toronto Unity Health, Toronto, ON Canada; 6 Division of Neurosurgery, Temerty Faculty of Medicine, University of Toronto, Toronto, ON Canada

**Keywords:** CNS cancer, Cancer metabolism

## Abstract

Glioblastoma accounts for nearly half of all primary malignant brain tumors in adults, and despite an aggressive standard of care, including excisional surgery and adjuvant chemoradiation, recurrence remains universal, with an overall median survival of 14.6 months. Recent work has revealed the importance of passenger mutations as critical mediators of metabolic adaptation in cancer progression. In our previous work, we identified a role for the epigenetic modifier ID-1 in temozolomide resistance in glioblastoma. Here, we show that ID-1-mediated glioblastoma tumourigenesis is accompanied by upregulation of one-carbon (1-C) mediated de novo purine synthesis. ID-1 knockout results in a significant reduction in the expression of 1-C metabolism and purine synthesis enzymes. Analysis of glioblastoma surgical specimens at initial presentation and recurrence reveals that 1-C purine synthesis metabolic enzymes are enriched in recurrent glioblastoma and that their expression correlates with a shorter time to tumor recurrence. Further, we show that the 1-C metabolic phenotype underlies proliferative capacity and temozolomide resistance in glioblastoma cells. Supplementation with exogenous purines restores proliferation in ID-1-deficient cells, while inhibition of purine synthesis with AICAR sensitizes temozolomide-resistant glioblastoma cells to temozolomide chemotherapy. Our data suggest that the metabolic phenotype observed in treatment-resistant glioma cells is a potential therapeutic target in glioblastoma.

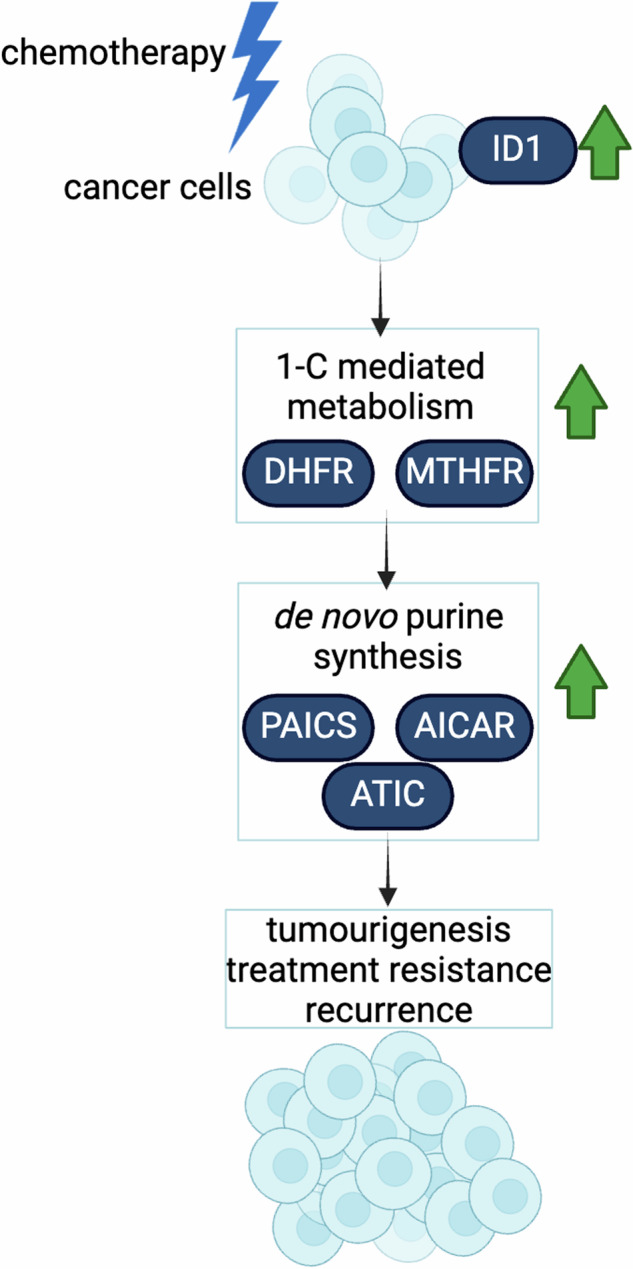

## Introduction

The nature of recurrence in glioblastoma and the general ineffectiveness of second-line therapies highlight the need for an improved understanding of the molecular characteristics of this disease. There is mounting evidence that anabolic pathways impact cancer cell metabolic reprogramming, drug resistance, proliferation, and stem-like properties, and these properties are often determined by passenger mutations-less critical mediators of cancer phenotype at the time of initial diagnosis. Additionally, reprogramming of cellular metabolism has been identified as a critical step in glial cell transformation during glioblastoma tumourigenesis [1, 2]. One-carbon (1-C) metabolism is a specific example of this phenomenon [3–7]. De novo purine synthesis and upregulation of the related 1-C metabolism pathway have been noted as a characteristic of less-differentiated stem and progenitor cells and brain tumor-initiating cells (BTICs) responsible for tumourigenesis [6, 8–10]. The de novo synthesis of both purines and pyrimidines relies on substrates and co-factors produced by upstream pathways, including 1-C metabolism. Dietary folate is first reduced tetrahydrofolate (THF) by dihydrofolate reductase (DHFR), for it to enter the folate cycle [11]. Once in the cycle, THF can bind methyl groups in various positions and act as an initial 1-C unit carrier for other biosynthetic pathways. 10-formyl-THF is a requisite substrate for de novo purine synthesis in mammalian cells and is produced from the reduction of 5,10-methyl-THF by methylenetetrahydrofolate dehydrogenase 2 like protein (MTHFD2/L) [11, 12]. De novo purine synthesis is a 10-step cytosolic reaction that results in the production of inosine monophosphate (IMP). IMP is further converted into guanosine monophosphate (GMP) via the activity of the enzymes inosine monophosphate dehydrogenase (IMPDH) and guanosine monosphosphate synthetase (GMPS), or adenosine monophosphate (AMP) via the activity of the enzyme adenylosuccinate synthase (ADSS) and adenylosuccinate lyase (ADSL). Deficiencies in 1-C metabolism, particularly THF and 10-formyl-THF, result in a deficiency of essential intermediates for purine synthesis, and an overall reduction in rates of de novo purine synthesis in a cell.

The transcriptional regulatory protein, an inhibitor of DNA-binding-1 (ID-1), is a key regulator of cell phenotype in cancer. ID-1 is a “medium-impact” passenger mutation: previous studies have shown the role of ID-1 in glioblastoma tumourigenesis to be inconstant and context specific[11–13]. ID-1 is enriched by treatment with temozolomide, a reflection of its role as a mediator of chemoresistance in glioblastoma [14]. ID-1 identifies relatively quiescent glioma-stem-like cells that are resistant to chemotherapy and able to serve as a repository for tumor recurrence[11, 14, 15].

ID-1 has been shown to regulate multiple metabolic pathways in other cancer types [16, 17]. These findings suggest that cancer cells, particularly stem-like cancer cells, maintain metabolic changes that promote sustainable growth and self-renewal, thereby achieving treatment resistance. Using a glioblastoma treatment resistance and disease recurrence model, we show that ID-1-mediated glioblastoma progression is accompanied by upregulation of 1-C mediated de novo purine synthesis and that the resulting metabolic phenotype underlies temozolomide resistance in glioblastoma.

## Results

### TMZ enriches ID-1 high cells with the metabolic phenotype of high 1-C mediated purine synthesis

**Fig. 1:**
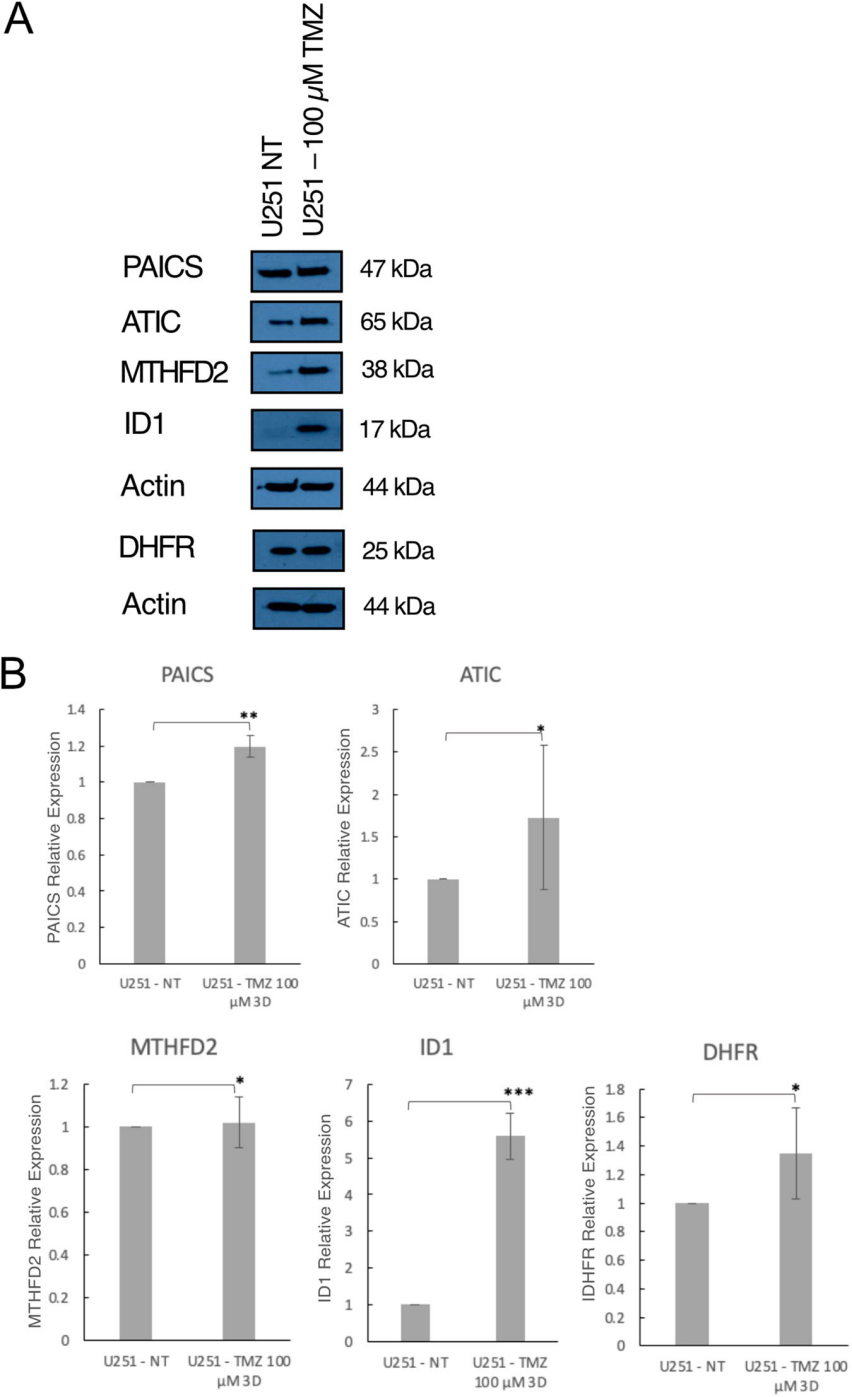
TMZ enriches for ID-1 high glioblastoma cells with increased expression of 1-C mediated purine synthesis enzymes. **A** Western blot analysis ratio of ID-1, DHFR, MTHFD2, PAICS, and ATIC expression in U251 glioblastoma cells after treatment for 3 days with 100 µM TMZ. **B** Quantitative densitometry ratios of ID-1, DHFR, MTHFD2, PAICS, and ATIC expression from western blot analysis. The data are normalized to untreated control and presented as mean ± SD. (*p ≤ 0.5, **p ≤ 0.05, ***p ≤ 0.01 by two-tailed *t*-test, *N* = 3).

In previous work, we have shown that temozolomide (TMZ) exposure results in enrichment of cells with high ID-1 expression [14]. Treatment with 100 µM of TMZ for three days resulted in an almost 3-fold increase in ID-1 expression in U251 glioblastoma cells. The increase in ID-1 expression was accompanied by an increase in the de novo purine synthesis enzymes, dihydrofolate reductase (DHFR), methylenetetrahydrofolate dehydrogenase 2 (MTHFD2), phosphoribosylaminoimidazole carboxylase (PAICS), and 5-Aminoimidazole-4-Carboxamide Ribonucleotide Formyltransferase/IMP Cyclohydrolase (ATIC) (Fig. 1A/B and Supplementary Fig. A/B). These data suggest that ID-1-high cells, which are enriched by TMZ therapy, are characterized by a metabolic phenotype of increased 1-C mediated purine synthesis.

### Changes in ID-1 expression mediate changes in one-carbon-mediated purine synthesis

**Fig. 2:**
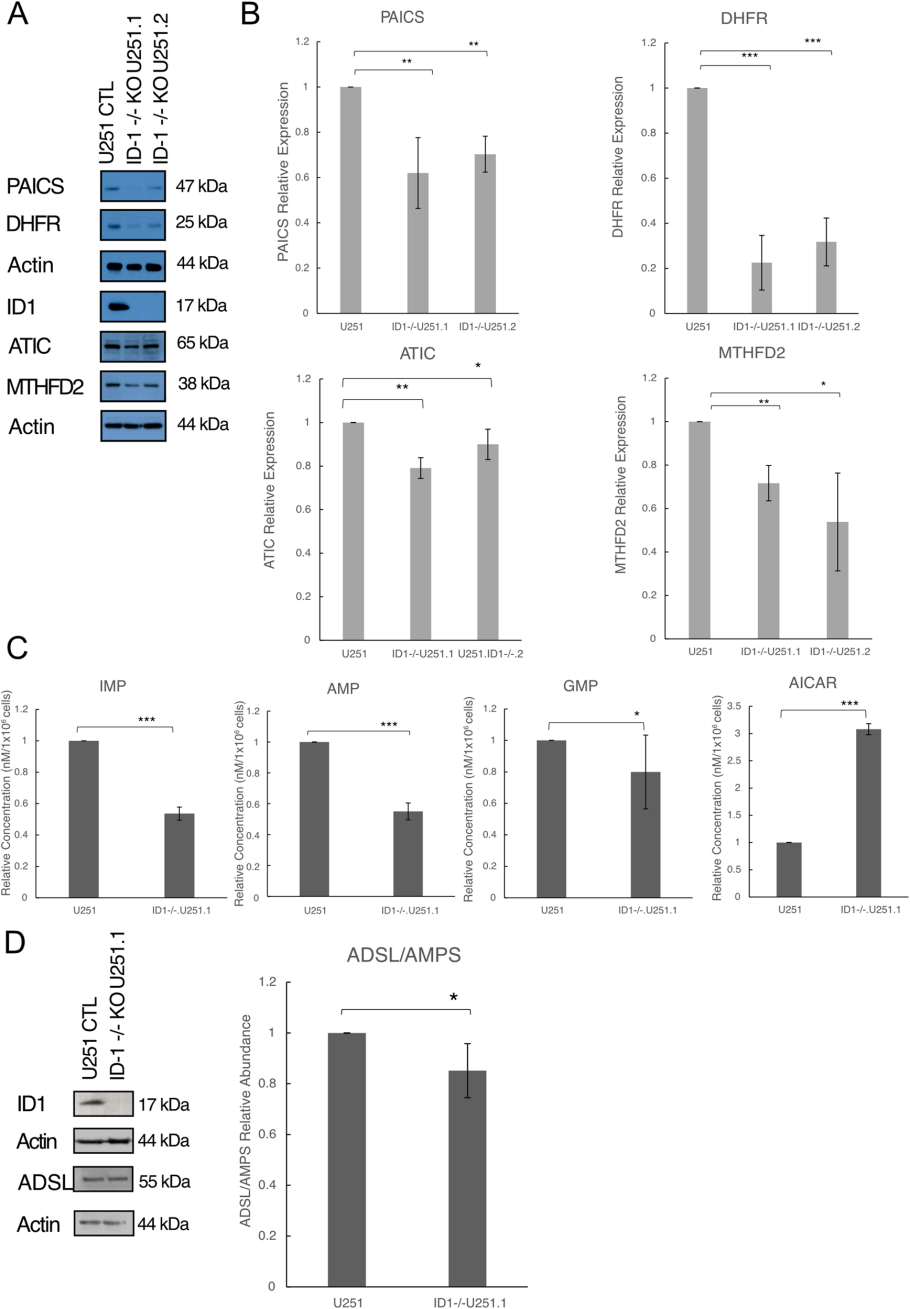
Reduction in ID-1 expression results in changes in 1-C mediated de novo purine synthesis. **A** Western blot analysis and quantitative densitometry ratios of 1-C metabolism enzymes DHFR and MTHFD2 in U251, ID-1^−/−^.U251.1, and ID-1^−/−^.U251.2 glioblastoma cells. **B** Western blot analysis and quantitative densitometry ratios of de novo purine synthesis enzymes PAICS and ATIC in U251, ID-1^−/−^.U251.1, and ID-1^−/−^.U251.2 glioblastoma cells. **C** LC-MS results showing a relative concentration of IMP, AMP, GMP, and AICAR in U251 and ID-1^−/−^.U251.1 cells. **D** Western blot analysis and quantitative densitometry ratios of ADSL/AMPS expression in U25 and ID-1^−/−^.U251.1 cells. All data are normalized to untreated control and presented as mean ± SD. (*p ≤ 0.5, **p ≤ 0.05, ***p ≤ 0.01 by two-tailed *t*-test, *N* = 3).

Using quantitative western blot analysis of lysates from CRSPR/Cas9-mediated glioblastoma ID-1-knockout cells (ID-1^−/−^U251.1, ID-1^−/−^U251.1) (U251-ID-1-null), we found lower expression of DHFR and MTHFD2 in U251-ID-1-null, compared to their parental controls (Fig. 2-A/B). Further, the expression of PAICS and ATIC was also significantly lower in ID-1 KO glioblastoma cells, compared to their parental controls (Fig. 2-A/B).

As deficits in 1-C metabolism would be predicted to result in the diminished availability of essential intermediates for purine synthesis, these data suggest that ID-1 KO glioblastoma cells harbor a metabolic phenotype characterized by purine deficiency. To test this hypothesis, we performed liquid chromatography–mass spectrometry (LC-MS) on polar metabolite extracts from U251 and U251-ID-1-null cells. U251-ID-1-null cells showed a significant reduction in the concentration of inosine monophosphate (IMP) (Fig. 2-C; 46% reduction, *p* = 1.5e-05), adenosine monophosphate (AMP) (Fig. 2-C; 45% reduction, *p* = 5.2e-05), and guanosine monophosphate (GMP) (Fig. 2-C 20% reduction, *p* = 0.1; Supplementary Fig. C/D). Additionally, we observed a 2-fold accumulation in the intermediate 5-aminoimidazole-4-carboxamide ribonucleotide (AICAR) (Fig. 2-C; *p* = 3.2e-05) in U251-ID-1-null cells. To determine if our finding of elevated AICAR levels was due to increased synthesis, we performed a western blot analysis for the enzyme ADSL, which is responsible for AICAR formation. ADSL levels were, in fact, slightly lower in the U251-ID-1-null cell line, compared to the parental line (Fig. 2-D; 15% reduction, *p* = 0.1; Supplementary Fig. E/F), suggesting that the increased concentration of AICAR was most likely due to the accumulation of this intermediate. These findings support the conclusion that the decrease in expression of one-carbon metabolic enzymes with ID-1 knockdown results in a reduction in purine synthesis in U251-ID-1-null cells.

### Chemical inhibition of ID-1 is associated with reduced 1-C-mediated purine synthesis

**Fig. 3:**
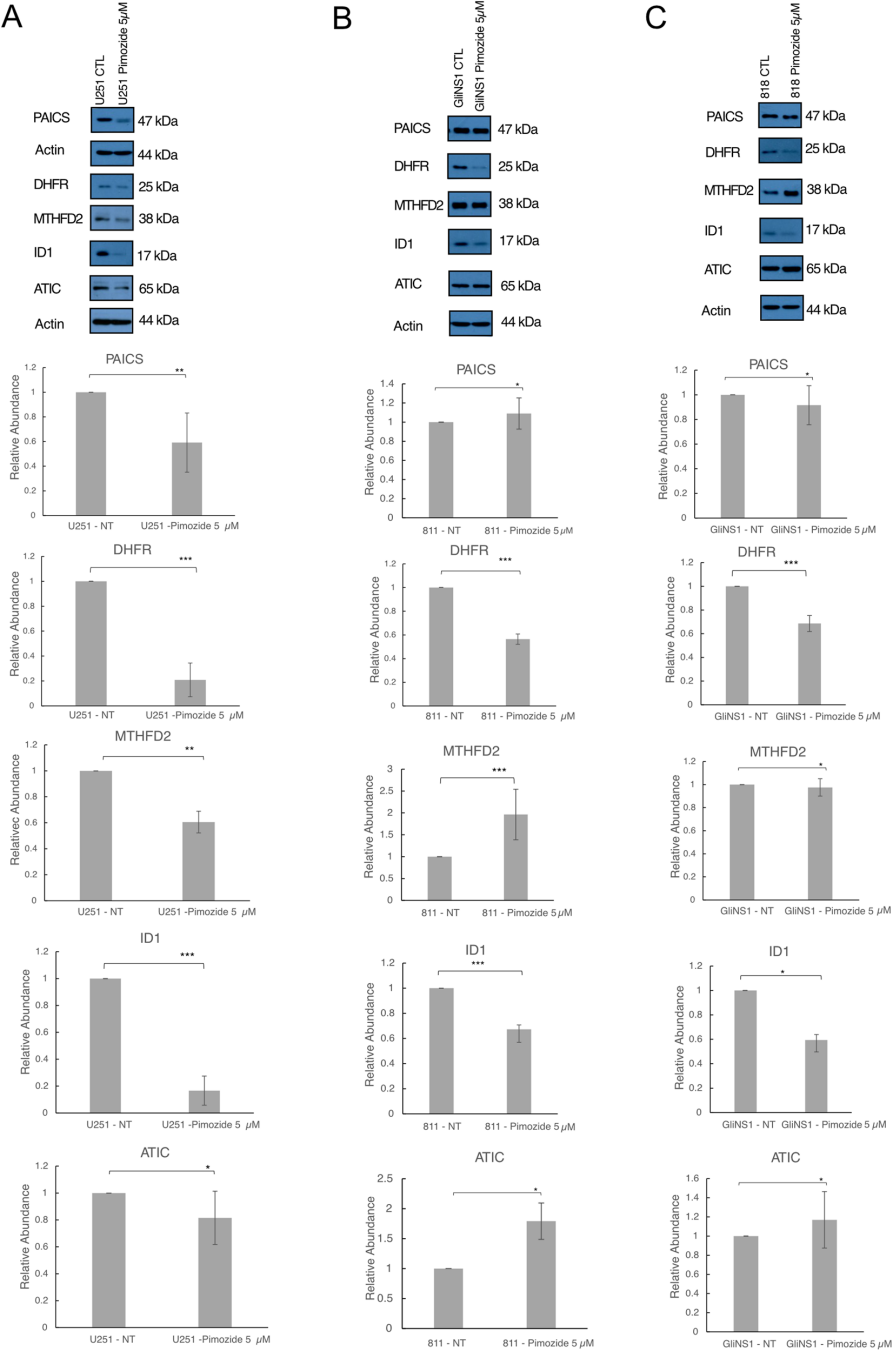
Chemical inhibition of ID-1 results in a reduction in expression 1-C metabolism and de novo purine synthesis enzymes. **A** Western blot analysis and quantitative densitometry ratios of ID-1, DHFR, MTHFD2, PAICS, and ATIC expression in U251 glioblastoma cells after 3-day treatment with 5 µM pimozide. **B** Western blot analysis and quantitative densitometry ratios of ID-1, DHFR, MTHFD2, PAICS, and ATIC expression in GliNS1 glioblastoma cells after 3-day treatment with 5 µM pimozide. **C** Western blot analysis and quantitative densitometry ratios of ID-1, DHFR, MTHFD2, PAICS, and ATIC expression in G811 glioblastoma cells after 3-day treatment with 5 µM pimozide. All data are normalized to untreated control and presented as mean ± SD. (*p ≤ 0.5, **p ≤ 0.05, ***p ≤ 0.01 by two-tailed *t*-test, *N* = 3).

To validate our findings that the reduction in metabolic enzyme expression in CRISPR-Cas9-mediated knockout was due to ID-1 depletion rather than off-target effects, we repeated these studies using pimozide, a chemical inhibitor of ID-1 [14]. Treatment of U251 cells with 5 µM pimozide resulted in a decrease in ID-1 expression from baseline (Fig. 3-A; *p* = 0.01; Supplementary Fig. G). Western blot analysis of the purine synthesis enzymes DHFR, MTHFD2, PAICS, and ATIC, showed a concomitant decrease in expression in pimozide-treated cells (Fig. 3-A and Supplementary Fig. G/H).

To confirm that our findings were biologically relevant to glioblastoma and not an artifact of a cancer cell line system, we repeated these studies in the glioma-stem cell (GSC) lines, GLiNS1 and G818 cells. Treatment of GLiNS1 and G818 cells with 5 µM pimozide resulted in a decrease in the expression of ID-1 and the purine synthesis pathway enzyme, DHFR, and an increase in the expression of ATIC (GliNS1), MTHFD2, and ATIC (G818), compared to non-treated cells. We speculated that the increase in ATIC and MTHFD2 could represent short-term changes to compensate for the reduction in purine synthesis resulting from inhibition of DHFR (Fig. 3-B/C).

### Reduced concentration of purine synthesis products limits the proliferative capacity of ID-1 KO glioblastoma cells

**Fig. 4:**
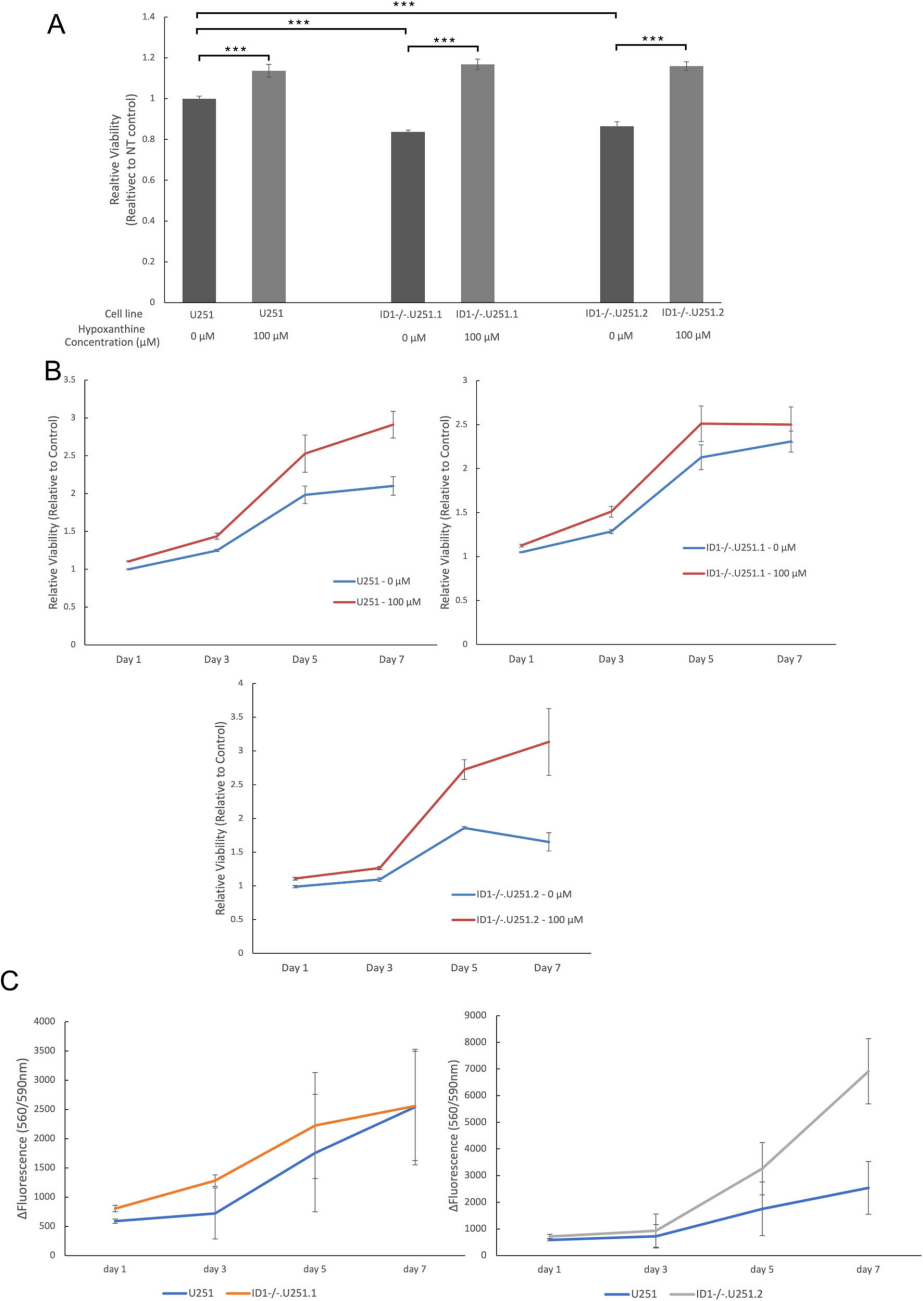
Exogenous purine supplementation restores proliferative capacity in U251-ID1-null glioblastoma cells. **A** Cell proliferation and viability analysis of U251, ID-1^−/−^.U251.1, and ID-1^−/−^.U251.2 cells after supplementation with 0 µM and 100 µM hypoxanthine for 3 days. The data are normalized to untreated control and presented as mean ± SD. **B** Cell proliferation and viability analysis of U251, ID-1^−/−^.U251.1, and ID-1^−/−^.U251.2 cells after supplementation with 100 µM hypoxanthine over the course of 7 days. Viability measurements were made at 1, 3, 5, and 7 days. The data are normalized to untreated control and presented as mean ± SD. **C** Relative change in viability and proliferation in hypoxanthine-supplemented and unsupplemented U251, ID-1^−/−^.U251.1, and ID-1^−/−^.U251.2 cells. The data are presented as mean ± SD change in fluorescence intensity. (*p ≤ 0.5, **p ≤ 0.05, ***p ≤ 0.01 by two-tailed *t*-test, *N* = 3).

To determine if purine synthesis inhibition with ID-1 KO resulted in a functional phenotype in glioblastoma cells, we performed a cell proliferation assay in U251-ID-1-null and U251 parental cells. U251-ID-1-null cells exhibited a significantly slower rate than U251 cells. To determine if this finding could be attributed to purine deficiency, U251-ID-1-null was then supplemented with exogenous hypoxanthine. Supplementation of U251-ID-1-null cells with 100 µM hypoxanthine supplementation for 3 days restored their proliferative rate to a level that superseded the parental cell line (Fig. 4-A). Supplementation of both U251-ID-1-null and parental U251 cells with hypoxanthine resulted in continuous proliferation at a rate that was significantly higher than their unsupplemented counterparts (Fig. 4-B), though the relative change in proliferation and viability was significantly higher in the U251-ID-1-null, compared to the parental U251 cells. (Fig. 4-C). Overall, these data suggest that the limitation of purine synthesis, limits the proliferative capacity of glioblastoma cells, and that purine salvage and the restoration of purine nucleotide concentrations rescues ID-1-KO cells to levels similar to their ID-1 intact counterparts.

### AICAR accumulation results in increased AMPK signaling and sensitizes U251 glioblastoma cells to TMZ

One of the intermediate metabolites in the purine de novo synthesis pathway is 5-aminoimidazole-4-carboxamide ribonucleoside (AICAR). AICAR has been shown to be a critical activator of the AMP-activated protein kinase (AMPK), which has been shown to reduce cancer cell growth in multiple cancer types, including glioblastoma [18–21]. AMPK activation by AICAR has been shown to induce cell death in childhood acute lymphoblastic leukemia cells [22], and sensitize prostate cancer cells to radiation therapy [23]. Of note, we found a 2-fold accumulation in AICAR in U251-ID-1-null cells, compared to their parental controls (Fig. 2-C).

**Fig. 5:**
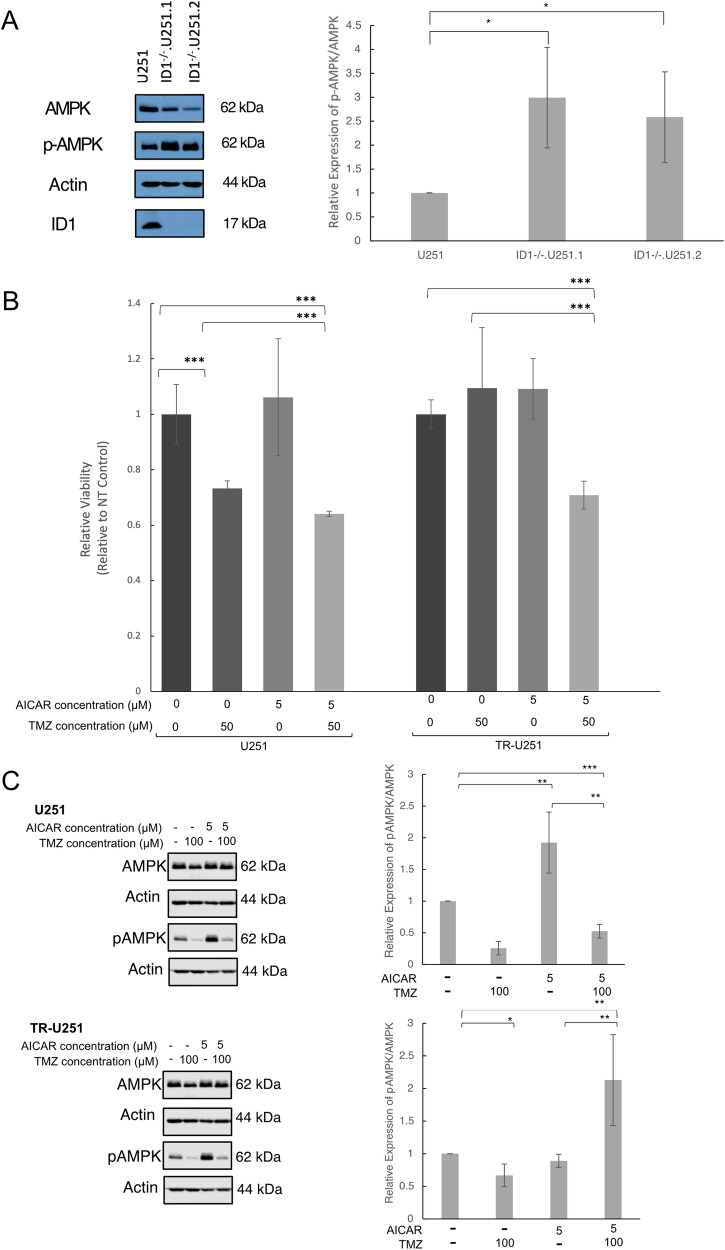
AICAR accumulation is associated with increased AMPK activation in U251-ID-1-null cells and sensitizes glioblastoma cells to TMZ chemotherapy. **A** Western blot analysis and quantitative densitometry ratios of AMPK and p-AMPK (Thr172) expression in ID-1^−/−^.U251.1, ID-1^−/−^.U251.2, and U251 cells. **B** Viability analysis of U251 and U251-TR cells treated with 5 µM AICAR, 50 µM TMZ, or a combination (5 µM AICAR + 50 µM TMZ) for 3 days**. C** Western blot analysis and quantitative densitometry ratios of AMPK and p-AMPK (Thr172) expression in U251 and U251-TR cells after treatment with 5 µM AICAR, 100 µM TMZ, and combined treatment. All data are normalized to untreated control and presented as mean ± SD. (*p ≤ 0.5, **p ≤ 0.05, ***p ≤ 0.01 by two-tailed *t*-test, *N* = 3).

To determine if AICAR can agonistically activate AMPK in glioblastoma, we performed a western blot analysis of U251-ID-1-null and parental U251 cells. The relative expression of p-AMPK to AMPK was significantly increased in both ID-1^−/−^.U251.1 and ID-1^−/−^.U251.2 cells, indicating increased activation of AMPK in ID-1 null cells compared to parental U21 cells (Fig. 5-A and Supplementary Fig. I).

We then sought to determine if AICAR could exert a cytotoxic effect on glioblastoma cells, analogous to that seen previously on pediatric ALL cells. Treatment with 5 µM AICAR had no effect on the viability of either U251 or U251-TR cells (Fig. 5-B). However, AICAR treatment did sensitize glioblastoma cells to the cytotoxic effects of temozolomide (TMZ). While treatment with 50 µM TMZ had no effect on the viability of U251-TR cells (Fig. 5-B), concomitant treatment with 5 µM AICAR resulted in a significant increase in U251-TR cell death (Fig. 5-B). These findings suggest that AICAR, at concentrations that are not cytotoxic individually, can sensitize glioblastoma cells to TMZ-induced cell death (Fig. 5-B). This effect may be, in part, mediated by the increased activation of AMPK in TR cells treated with a combination of AICAR and TMZ. The expression of p-AMPK relative to AMPK was significantly increased in U251-TR cells treated with concomitant TMZ and AICAR (Fig. 5-C and Supplementary Fig. J).

### Glioblastoma recurrence is associated with an increase in de novo purine synthesis enzyme expression

In previous work, we had shown that patients harboring tumors with a greater than 2-fold increase in ID-1 expression following TMZ chemotherapy had a shorter progression-free survival (PFS) [14].

**Fig. 6:**
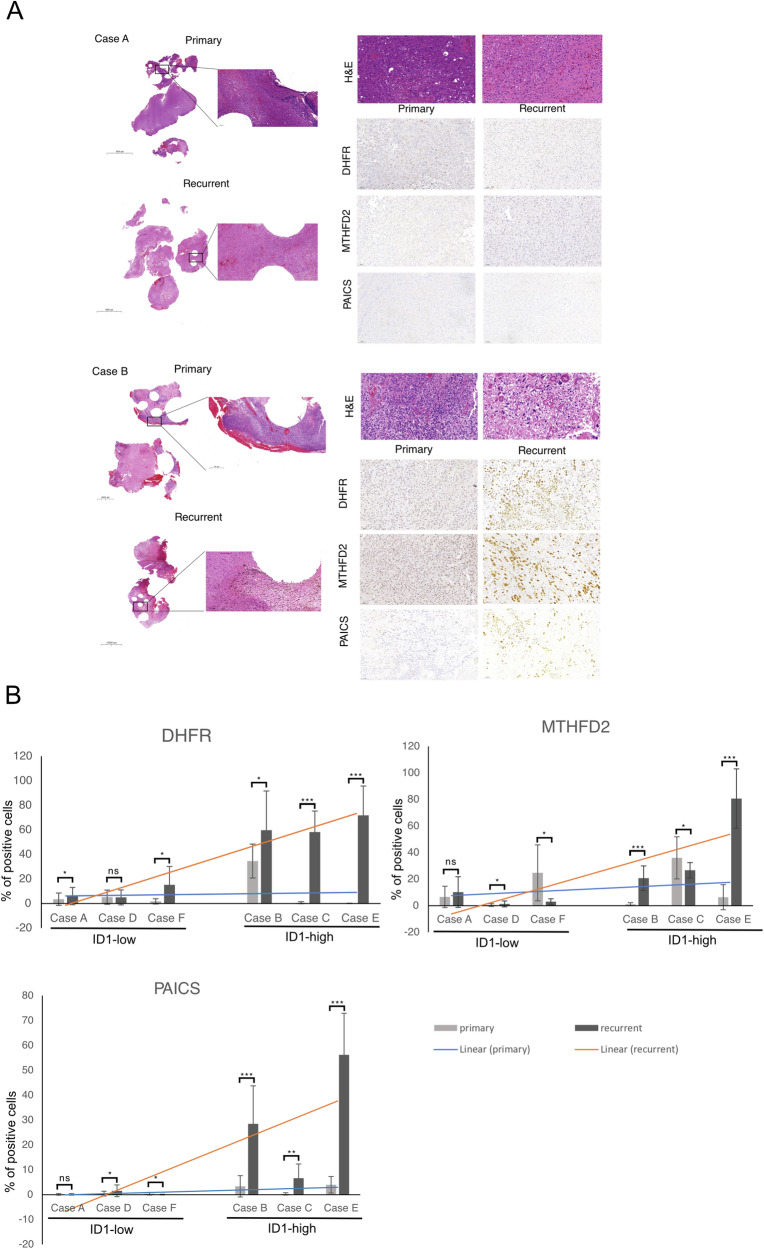
TMZ chemotherapy enriches for ID-1 and 1-C mediated purine synthesis enzymes in glioblastoma patients. ID-1 and metabolic enzyme expression studies were performed on samples with pathologically confirmed glioblastoma recurrence following temozolomide chemotherapy. ID-1-high: cases with more than a 2-fold increase in ID-1-expression after recurrence, ID-1-low: cases with less than 2-fold increase in ID-1-expression after recurrence. **A** Representative images from IHC analysis of primary and recurrent glioblastoma cases from ID-1-low (case A) and ID-1-high (case B) tumors stained for PAICS, MTHFD2, and DHFR. **B** Percentage of cells staining positive for DHFR, MTHFD2, and PAICS from ID-1-low (case A, D, and F) and ID-1-high (case B, C, and E) cases. Blue trendline represents primary cases, orange trendline represents recurrent cases. All data are presented as absolute mean percentage values of 6 fields of view per case ± SD. (ns = non-significant, *p* > 0.5, *p ≤ 0.5, **p ≤ 0.05, ***p ≤ 0.01 by two-tailed *t*-test).

To determine if tumor recurrence in glioblastoma patients who have undergone TMZ chemotherapy is associated with an increase in de novo purine synthesis enzyme expression, we performed IHC to determine the expression of metabolic enzymes DHFR, MTHFD2, and PAICS in ID-1-high patients. We found a significant relative increase in the expression of de novo purine synthesis enzymes in recurrent tumors with high ID-1 expression, compared to ID-1-low tumors (Fig. 6-A/B). These findings suggest that the metabolic phenotype of increased 1-C mediated purine synthesis enriched by TMZ chemotherapy mediates chemoresistance and a quiescent stem-like state that allows the initiation of tumor recurrence and may underly the shorter PFS seen in patients with ID-1-high recurrent glioblastoma.

## Discussion

We found that glioblastoma tumourigenesis in an ID-1 KO model of glioblastoma treatment resistance and disease recurrence is accompanied by upregulation of 1-C mediated de novo purine synthesis. The accumulation of AICAR and the reduced concentration of de novo purine synthesis products in ID-1^−/−^ U251 glioblastoma cells supports a conclusion that the rate of de novo purine synthesis is lower in ID-1^−/−^ cells compared to wild-type. Our results are supported by studies indicating that alterations in 1-C metabolism—and MTHFD2 expression in particular—affect the rates of purine synthesis [5]. A recent study by Nakamizo et al. also shows that the purine synthesis pathway is upregulated in glioblastoma tumors, and purine salvage is downregulated [24]. It is known that despite the extracellular abundance of certain metabolites, many cancers rely on actively synthesizing their own stores [25]. Similar to this, we suggest that glioblastoma cells rely on the endogenous production of nucleotides for survival and the upregulation of purine and pyrimidine synthesis pathways, and associated pathways such as 1-C metabolism, which provide them with a proliferative advantage.

ID-1 maintains a state of less-differentiation and rapid proliferation in cells, however, the role of ID-1 in tumourigenesis has been shown to be context specific [11]. We suggest that a critical element of the chemoresistant phenotype of ID-1 high glioblastoma cells, which represent relatively quiescent glioma-stem-like cells, is the increased expression of enzymes involved in anabolic pathways. Tumor cells have a fundamental requirement for purine nucleotides in order to maintain proliferation and employ a variety of strategies to maintain their nucleotide pools [5]. For example, the expression of enzymes involved in 1-C-mediated purine synthesis is increased in a variety of tumor types [4, 5, 8, 26–28]. Based on our findings, purine synthesis enzyme expression and concentrations of purine nucleotide precursors are both reduced in ID-1^−/−^ glioblastoma cells, and this change in the metabolic phenotype of the cell consequently limits the proliferation capacity of the cells. Stem cells (like cancer cells) employ metabolic programs to maintain the biomass required for self-renewal and proliferation. We hypothesize that as a passenger mutation, ID-1 expression in glioblastoma cells promotes 1-C mediated purine synthesis, and thereby facilitates the maintenance of stem-like characteristics and proliferative capacity. Our results accord with other studies that show reductions in 1-C metabolism ultimately result in diminished proliferative capacity [5].

While supplementation with hypoxanthine showed a rescue effect on proliferation in ID-1^−/−^.U251.1 and ID-1^−/−^.U251.2 cells, hypoxanthine supplementation had a less significant effect on proliferation in WT U251 cells. We suspect that high levels of purine synthesis in ID-high cells result in substrate saturation. Reduction in the rate of a metabolic process is often accompanied by an accumulation of metabolic intermediates. Our results, similar to other groups, show that decreases in the rate of de novo purine synthesis result in the accumulation of AICAR [4, 8]. We show that the accumulation of AICAR in glioblastoma cells is due to impairment of the mechanisms involved in the conversion of AICAR to AMP upon ID1 knockout, as evidenced by the reduction in ADSL expression in ID-1^−/−^ cells. In fact, the accumulation of AICAR may even contribute to a negative feedback mechanism, further reducing de novo purine synthesis in ID-1^−/−^ cells. Furthermore, our results show that the accumulation of AICAR in ID-1^−/−^ glioblastoma cells is accompanied by increased AMPK/p-AMPK signaling.

Based on the results presented, we suggest that the AICAR-mediated agonistic increased activation of AMPK in ID-1^−/−^ cells creates a synergistic effect, resulting in the reduction of macromolecule synthesis to an extent where the proliferative capacity of the cell is significantly reduced. As suggested by the literature, other metabolic pathways can also be affected by AMPK signaling—the metabolic reprogramming observed in these cells might involve multiple processes that this study has not touched upon. The reduced levels of enzymes involved in de novo purine synthesis observed in the ID-1^−/−^ glioblastoma cells might, in fact, be exacerbated by the activation of AMPK. It stands to be determined whether the change in AMPK signaling is directly mediated by ID-1. Further studies are required to elucidate the regulatory role of AMPK signaling in the metabolic reprogramming of ID-1-high glioblastoma cells.

In accordance with other studies, we show that AICAR can sensitize treatment-resistant cells to chemotherapy with TMZ [1, 4, 29]. AICAR-mediated metabolic changes can affect the availability of macromolecules for proliferation and DNA repair. The reduced availability of nucleotides would leave cells more vulnerable to the effects of chemotherapy-induced DNA damage. Additionally, AICAR-enhanced TMZ cytotoxicity may be related to signaling pathways, including p21 or p53 pathways, which are activated by AICAR itself [22, 30]. In combination with the cytotoxic effects of TMZ, the cytotoxicity enhanced by AICAR-mediated AMPK activation can increase overall rates of cancer cell death and treatment effectiveness. Further study is needed to define the mechanism of AICAR-mediated TMZ sensitivity in treatment-resistant glioblastoma cells. It is important to note that AICAR was a less effective adjunct in the sensitization of ID-1^−/−^.U251 cells to TMZ, compared to U251-TR cells. This might be attributable to the high endogenous levels of AICAR in ID-1^−/−^.U251 cells saturating AICAR-mediated signaling. If so, this finding would support our hypothesis that the metabolic phenotype seen in ID-1 expressing glioblastoma cells underlies treatment resistance in glioblastoma.

In our prior work, we found that ID-1-high cells are enriched in glioblastoma by TMZ chemotherapy [11, 14]. In this study, we find that TMZ treatment also results in an enrichment of cells expressing higher levels of 1-C-mediated purine synthesis enzymes. Cases with more than a 2-fold increase in ID-1-expression after recurrence were observed to also harbor higher expression of 1-C mediated purine synthesis enzymes. High rates of 1-C-mediated purine synthesis prepare a cell metabolically for rapid division and proliferation [31, 32]. Higher nucleotide availability also renders a cell more capable of DNA damage repair, allowing the cell potential for resistance towards TMZ cytotoxicity [33]. Increased ID-1 expression in glioblastoma cells mediates chemoresistance and a quiescent stem-like state that allows these cells to initiate tumor recurrence. We have shown that the expression of ID-1 also mediates metabolic reprogramming and a reliance on 1-C-mediated purine synthesis for proliferation and chemoresistance. Reliance on this metabolic phenotype may, in turn, increase vulnerability to inhibition of metabolic programs. Targeting 1-C metabolism and purine synthesis in cancer cells has gained much recent interest. Further elucidation of the role of 1-C metabolism in glioblastoma tumourigenesis may provide a basis for the clinical consideration of these novel metabolic treatment strategies in the treatment of glioblastoma.

The data presented identify a metabolic phenotype underlying treatment resistance in ID-1-expressing glioblastoma cells. While further research is required to detail the interactions between ID-1 regulation, AMPK signaling, and metabolic reprogramming in glioblastoma cells, our data suggests that reprogramming of 1-C mediated purine synthesis plays a role in glioblastoma cell proliferation and treatment resistance. This study, in addition to further research, can help clarify the mechanisms through which ID-1, as a passenger mutation, mediates chemoresistance in glioblastoma cells.

## Methods

### Cell culture

The glioblastoma cell line U251 was obtained from the American Type Culture Collection (ATCC). ID-1-knockout (ID-1^−/−^.U251.1, ID-1^−/−^.U251.2) cell lines were produced using a CRISPR/Cas9 system as previously described (Sachdeva et al., [11]). U251 wild type and ID-1^−/−^ cell lines were grown in Dulbecco’s modified eagle media (DMEM, Cat#319-050-CL, Wisent, ST-BRUNO, Quebec Canada) with 10% Fetal Bovine Serum (FBS) (Cat#920-040, Wisent) and 1% penicillin/streptomycin (Cat# 450-200-EL, Wisent).

Temozolomide-resistant U251 cell lines (U251-TR) were generated by treating U251 cells with increasing concentrations of Temozolomide (TMZ) (10 µmol/L to 200 µmol/L) biweekly. When passaged, 100 µM TMZ was added to the cell media to maintain the treatment-resistant phenotype in these cells.

The GSC line G818 (gift from Dr. Frederick Lang, Department of Neurosurgery, MD Anderson Cancer Center, Houston, TX) and GliNS1 (a gift from Dr. Peter Dirks, Hospital for Sick Children, Toronto, Canada) were cultured as neurospheres in DMEM F-12 (Cat# 319-075-CL, Wisent) supplemented with 2 mmol/L l-glutamine (Cat# 25030081, Thermo Fisher Scientific Inc), 1× antibiotic/antimycotic (Cat# 15240062, Thermo Fisher Scientific Inc), 2% B27 supplement (Cat# 17504044, Thermo Fisher Scientific Inc), 20 ng/mL human epidermal growth factor (hEGF, Cat#9644, Sigma), and 20 ng/mL human basic fibroblast growth factor (hFGF, Cat# F5542, Sigma).

All cell lines were cultured at 37 °C in a humidified incubator at 5% CO_2_. Cell lines were routinely tested for *Mycoplasma* contamination using PCR.

### Cell viability assay

Cell viability was assessed using the Promega CellTitre-Blue^®^ Cell Viability Assay (Cat# G8080, Promega). 1 × 10^3^ to 2 × 10^3^ cells were seeded in 96-well plates (Sarstedt Inc, 83.3924), in eight replicates. The cells were treated according to the requirements of each experiment. After the determined period of incubation, the CellTitre-Blue® reagent was added to each well at an equivalent of 5% of the total cell culture volume/well. After a 3-h incubation in the dark at 37 °C and 5% CO_2_, fluorescence was measured with the SpectraMax Gemini EM (Molecular Devices) fluorescence plate reader with excitation wavelength set to 560 nm and emission wavelength set to 590 nm. The fluorescence values generated were collected by the SoftMax Pro software and analyzed to measure the relative viability between different treatment groups.

### Western blotting

Membranes were blotted with antibodies for ID-1 (sc-488, Santa Cruz), AMPK α (Cat#5831, Cell Signalling), p-AMPK α (Cat#2535, Cell Signalling), AMPS (Cat#ab154182, Abcam), ATIC (Cat #PA5-82741, Invitrogen), DHFR (Cat#sc-377091, Santa Cruz), HPRT (Cat#ab109021, Abcam), MTHFD2 (Cat# ab151447, Abcam), PAICS (Cat# PA5-57452, Invitrogen), or β-Actin (Cat#4970, Cell Signalling). Bound antibodies were detected with horseradish peroxidase-linked anti-mouse or anti-rabbit IgG (Cell Signaling Technology), followed by ECL (PerkinElmer). Unprocessed western blot films can be accessed in the supplementary materials included with this publication.

### Immunohistochemistry

Patient tissues and clinical information were obtained with written informed consent from St. Michael’s Hospital CNS Biobank, as approved by the Research Ethics Boards at St. Michael’s Hospital (Toronto, Canada) and the Hospital for Sick Children. ID-1 and metabolic enzyme expression studies were performed on samples with pathologically confirmed recurrence following temozolomide chemotherapy. Paraffin-embedded blocks were cut into 5-μm sections and dewaxed in xylene, followed by rehydration in a standard alcohol series (90, 70, 50%). Antigen retrieval was achieved by 20 min of pressure cooking in citrate buffer (pH 6.0), followed by blocking for 10 min with Universal Blocking Buffer (Cat# X0909, Dako). The slides were then incubated with antibodies for DHFR, MTHFD2, and PAICS overnight at 4 °C. Detection used biotinylated secondary antibodies for 1 h, the ABC Reagent Kit (Vector Labs), and DAB chromogen (Vector Labs). Slides were washed with PBS three times after each step. Sections were then dehydrated using increasing concentrations of ethanol (50, 70, 90%), followed by a brief washing in xylene. Finally, the slides were mounted in Permount (Thermo Fisher Scientific). Immunoexpression of proteins of interest was quantified as percentage positive cells with QuPath Image Analysis Software [34, 35]. All data were presented as absolute mean percentage values of 6 fields of view per case ± SD.

### Targeted quantification of metabolite concentrations

U251, ID-1^−/−^.U251.1, or ID-1^−/−^.U251.2 cells (2–5 × 10^6^ cells/plate) were seeded in 150 × 25 mm cell culture dishes (Falcon) in DMEM with 10% FBS and 1% P/S media, then serum-deprived after 24 h. Cells were grown for 72 h until all plates reached 95–100% confluency. Metabolites were extracted using −20 °C extraction buffer (40% methanol, 40% acetonitrile, 20% H_2_O, 0.1 mM Formic Acid). The samples were prepared for LC-MS analysis as described by Rabinowitz and Kimball (2007). The extracts were dried using a non-heat vacuum dryer (Thermo Scientific, Savant RVT5105 Refrigerated Vapor Trap, and Savant SPD121P SpeedVac Concentrator). The samples were submitted to the BioZone at the University of Toronto for LC-MS analysis using an Exactive orbitrap mass spectrometer equipped with an electrospray ionization source and a Hypersil Gold C18 column (50 mm × 2.1 mm, 1.9 µm particle size; Thermo Scientific) or a Luna NH2 column (150 mm × 2 mm, 3 µm; Phenomenex).

### Exogenous supplementation of purine metabolism with hypoxanthine

2 × 10^3^ U251, ID-1^−/−^.U251.1. ID-1^−/−^.U251.2, or U251-TR cells, were seeded in 96 plate wells (Sarstedt Inc) in 200 µL of DMEM with 1%FBS and 1% P/S media. Hypoxanthine (Sigma-Aldrich) was resuspended in a 2:1 Formic-acid:Water solution (Sigma-Aldrich), as per the manufacturer’s instructions, to produce a 100 mM stock. Cells were supplemented with 0 µM, 100 µM, and 200 µM dilutions of stock hypoxanthine into DMEM containing 1% FBS and 1% P/S for 1, 3, 5, or 7 days.

### AICAR and temozolomide combined treatment

2 × 10^3^ U251, ID-1^−/−^.U251.1, ID-1^−/−^.U251.2, and U251-TR cells were seeded in 96 plate wells (Sarstedt Inc) in DMEM (1% FBS, 1% P/S media). Cells were treated with 0 µM, 5 µM, 15 µM, 20 µM, and 30 µM AICAR diluted in DMEM (1% FBS, 1% P/S) in combination with 0 µM, 25 µM, 50 µM, and 100 µM TMZ diluted in DMEM (1% FBS, 1% P/S).

### Statistical analysis

All experiments were performed in three biological and technical replicates. Mean and standard deviation (SDV) were used where appropriate. The data was assessed for normality using normality distribution assumptions. Two-tailed *t*-tests and Linear regression analyses were used. Statistics were completed with Microsoft Excel 2013. *, **, ***, **** denotes significance levels of *p* ≤ 0.05, *p* ≤ 0.01, *p* ≤ 0.001, *p* ≤ 0.0001, respectively. Error bars represent SDV.

All methods in this study were performed in accordance with the relevant guidelines and regulations, and in accordance with REB approval from our research institutions.

## Supplementary information

Supplementary Materials _Western Blot original films
