# Supplementary material for: One-carbon-mediated purine synthesis underlies temozolomide resistance in glioblastoma
Source: Cell Death Dis. 2024 Oct 25;15(10):774. doi: 10.1038/s41419-024-07170-y (PMC11511812; doi:10.1038/s41419-024-07170-y)
Supplement: Supplementary file 1 — Supplementary Materials _Western Blot original films [file 41419_2024_7170_MOESM1_ESM.pdf]

Supplementary Material

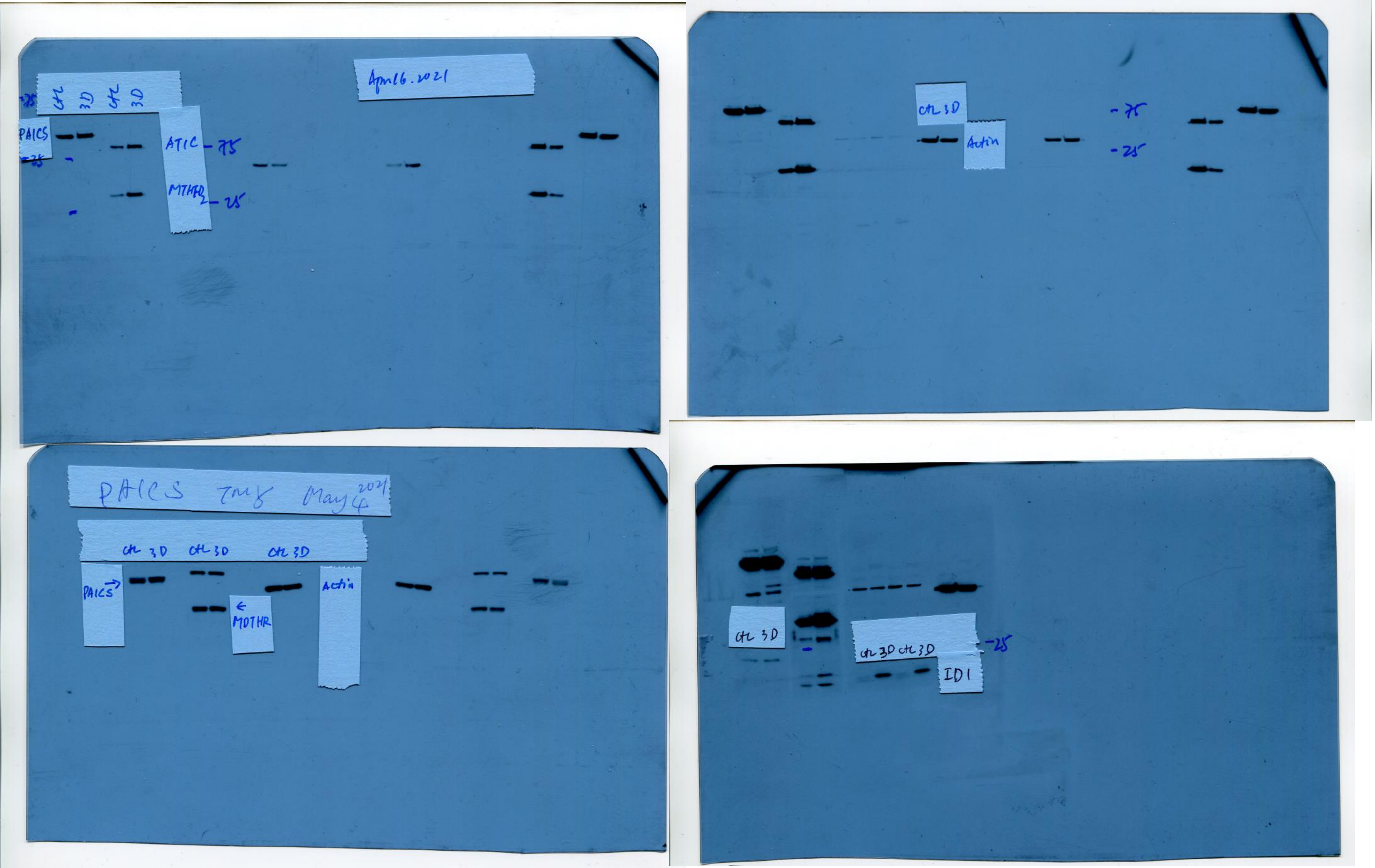

Supplementary figure A. Original western blot films pertaining to manuscript Figure 1.

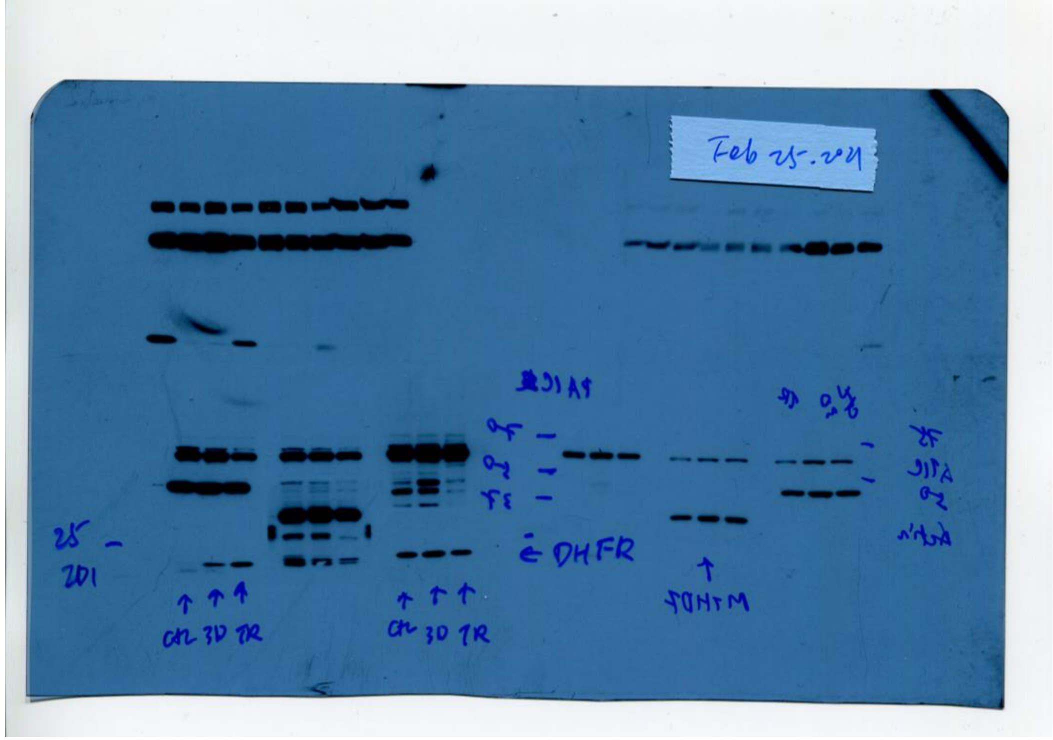

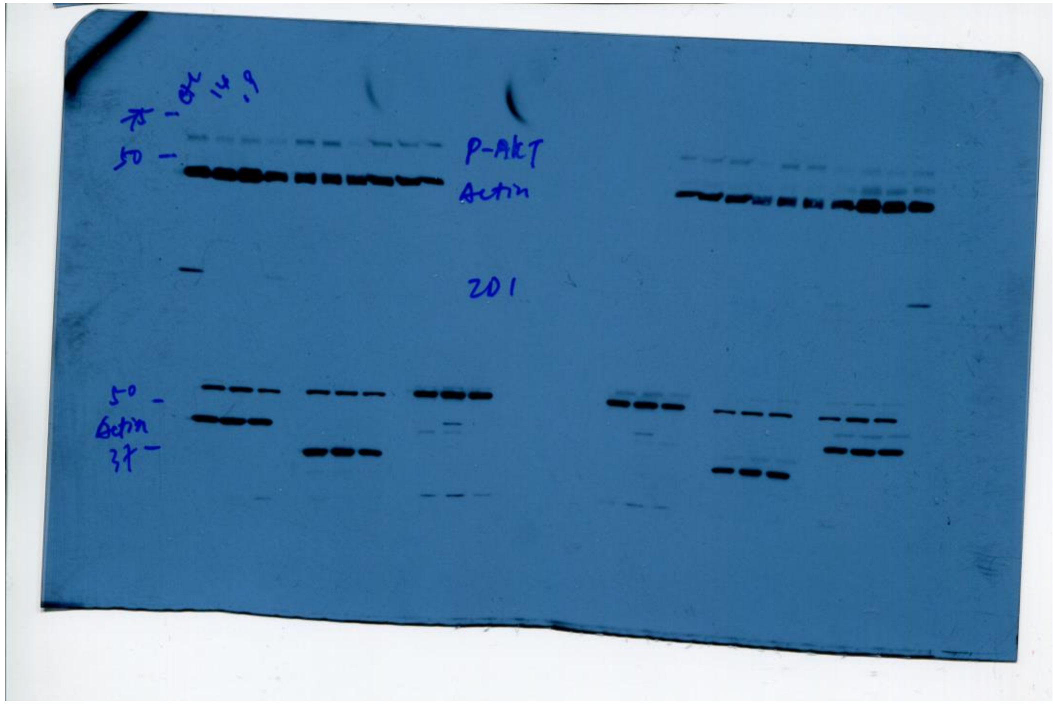

Supplementary figure B. Original western blot films pertaining to manuscript Figure 1.

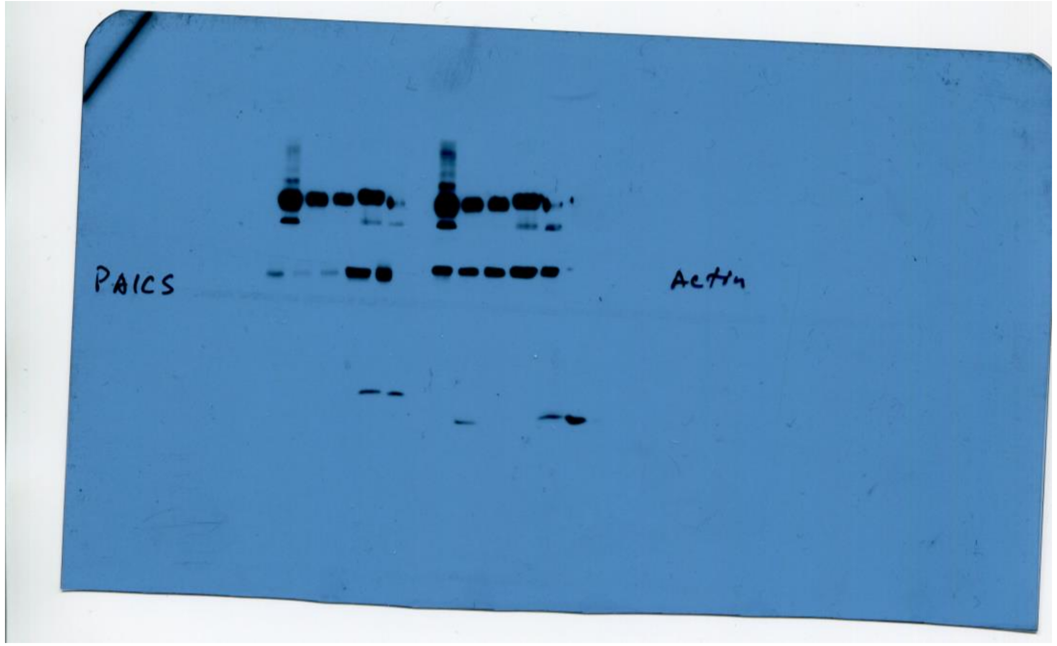

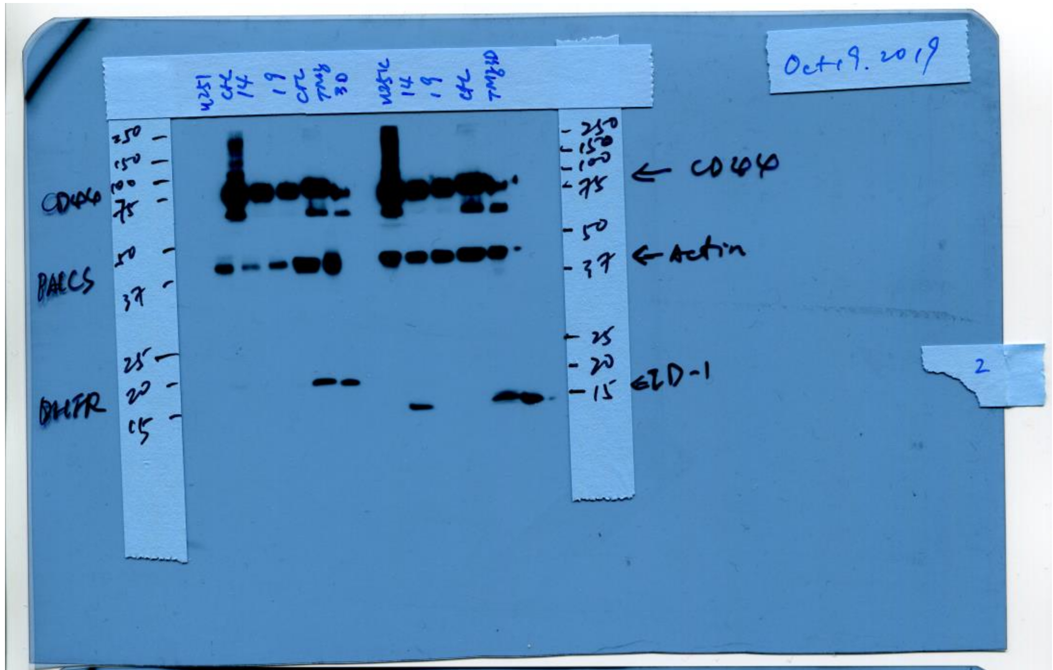

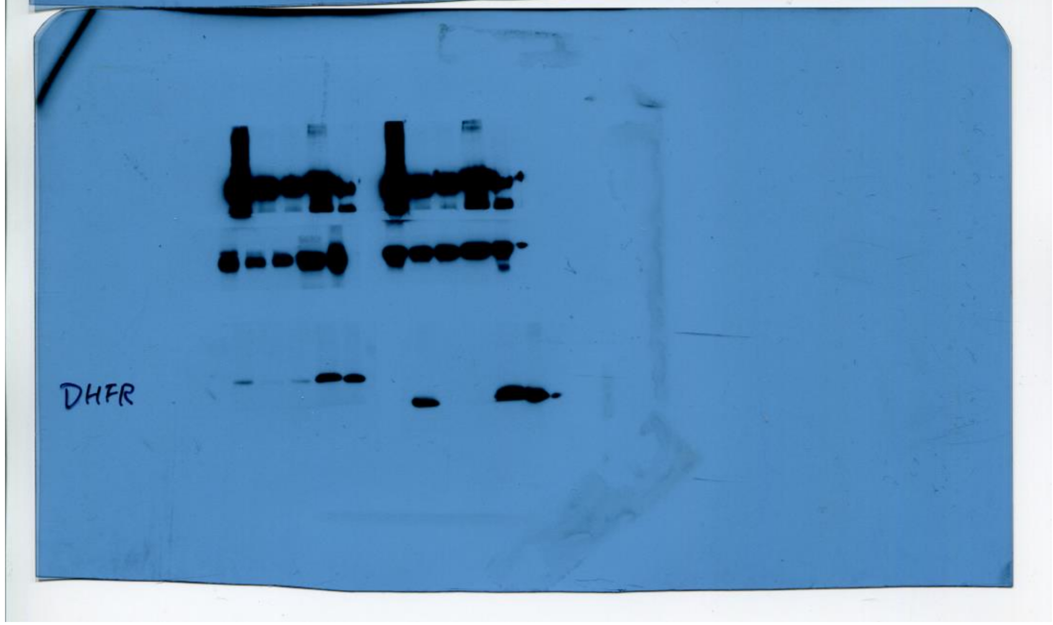

Supplementary figure C. Original western blot films pertaining to manuscript Figure 2.

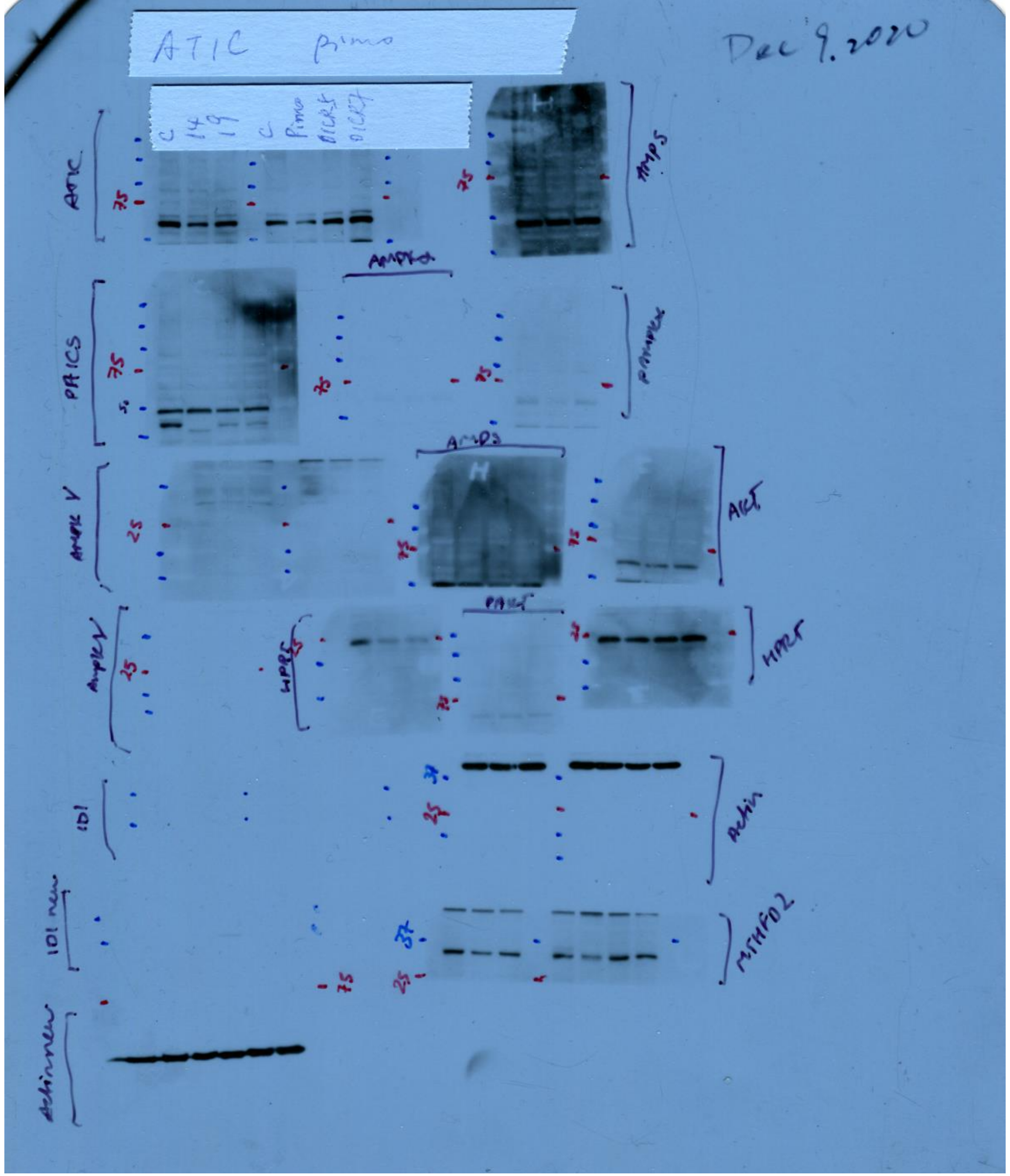

Supplementary figure D. Original western blot films pertaining to manuscript Figure 2.

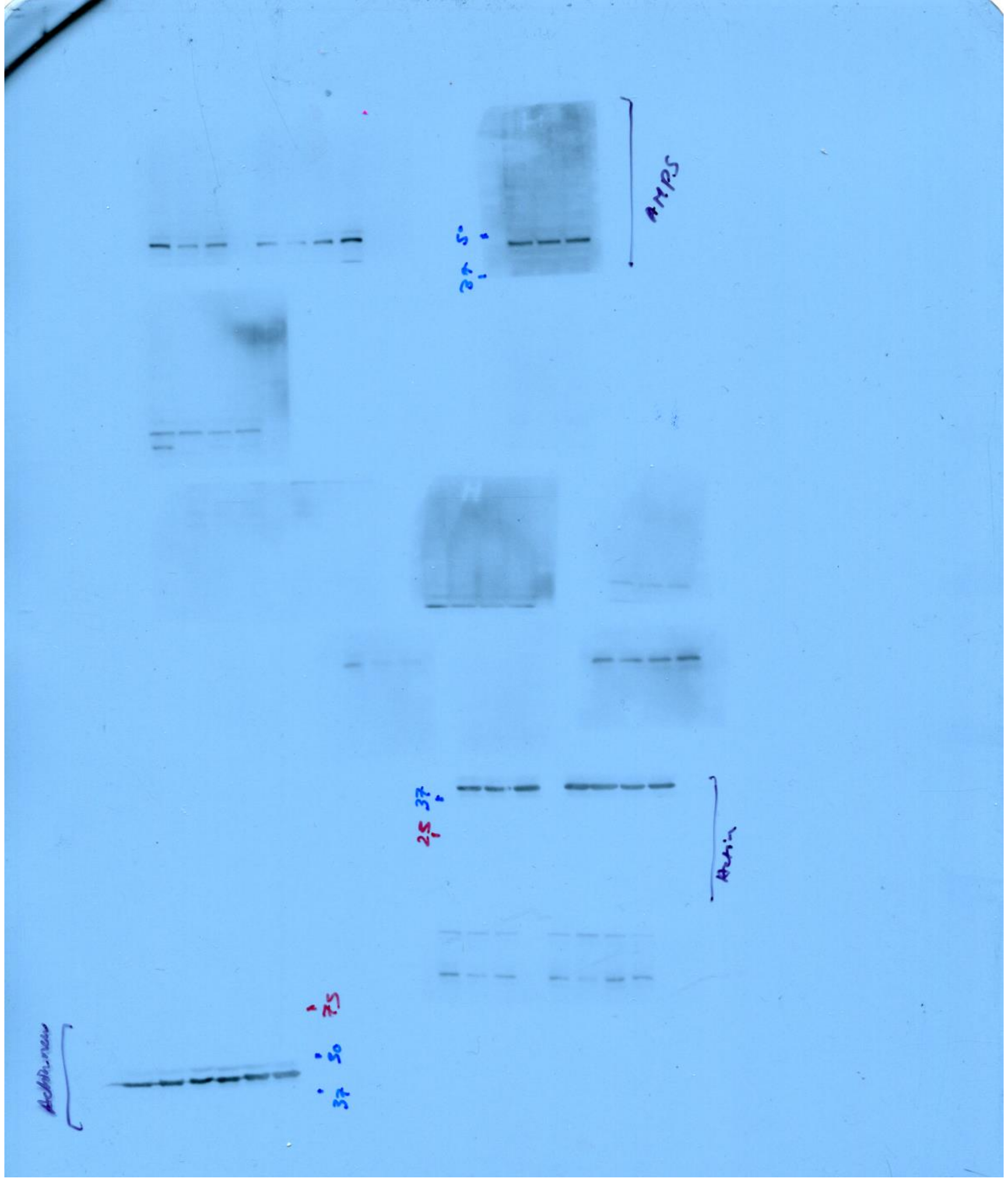

Supplementary figure E. Original western blot films pertaining to manuscript Figure 2-D.

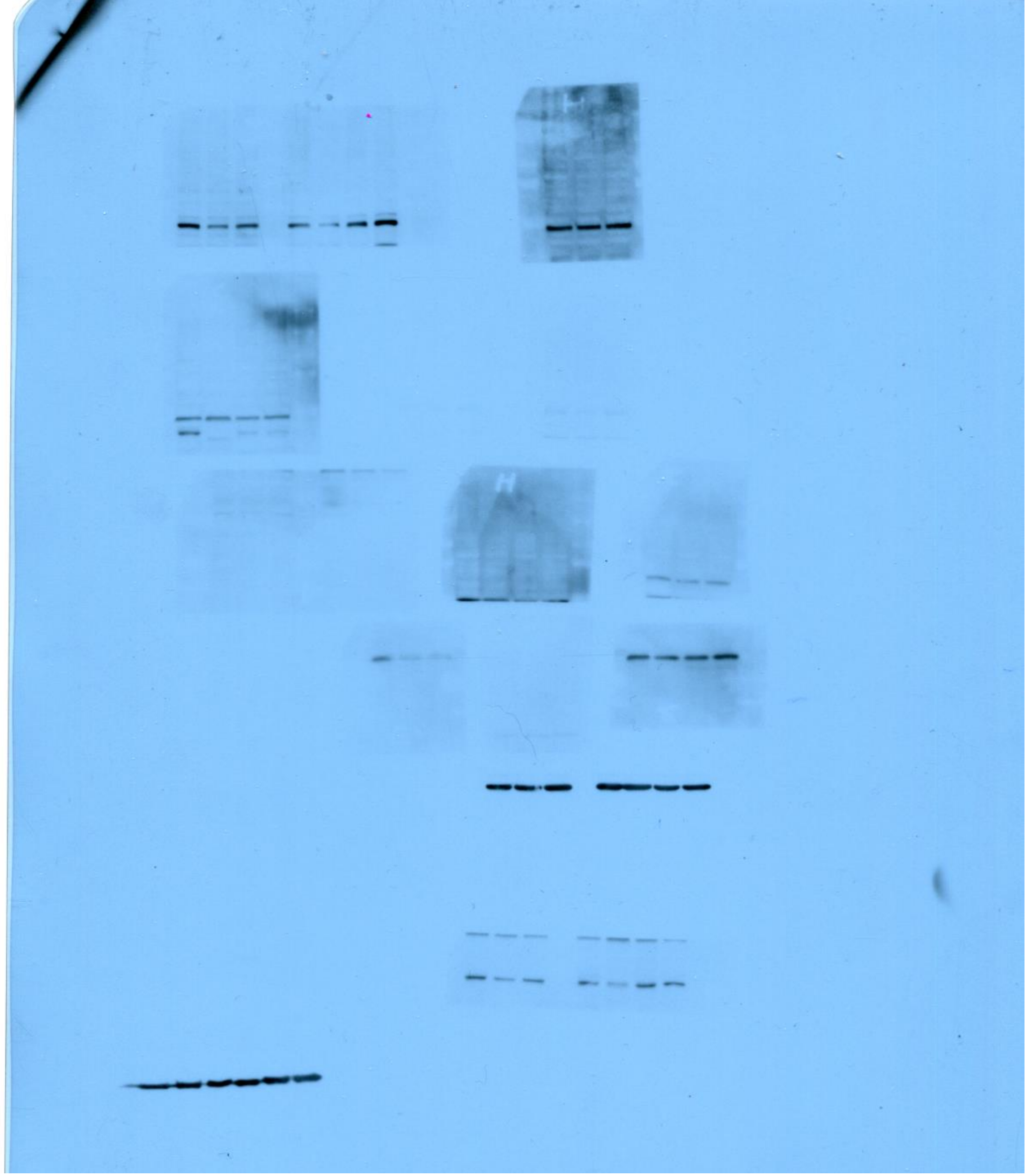

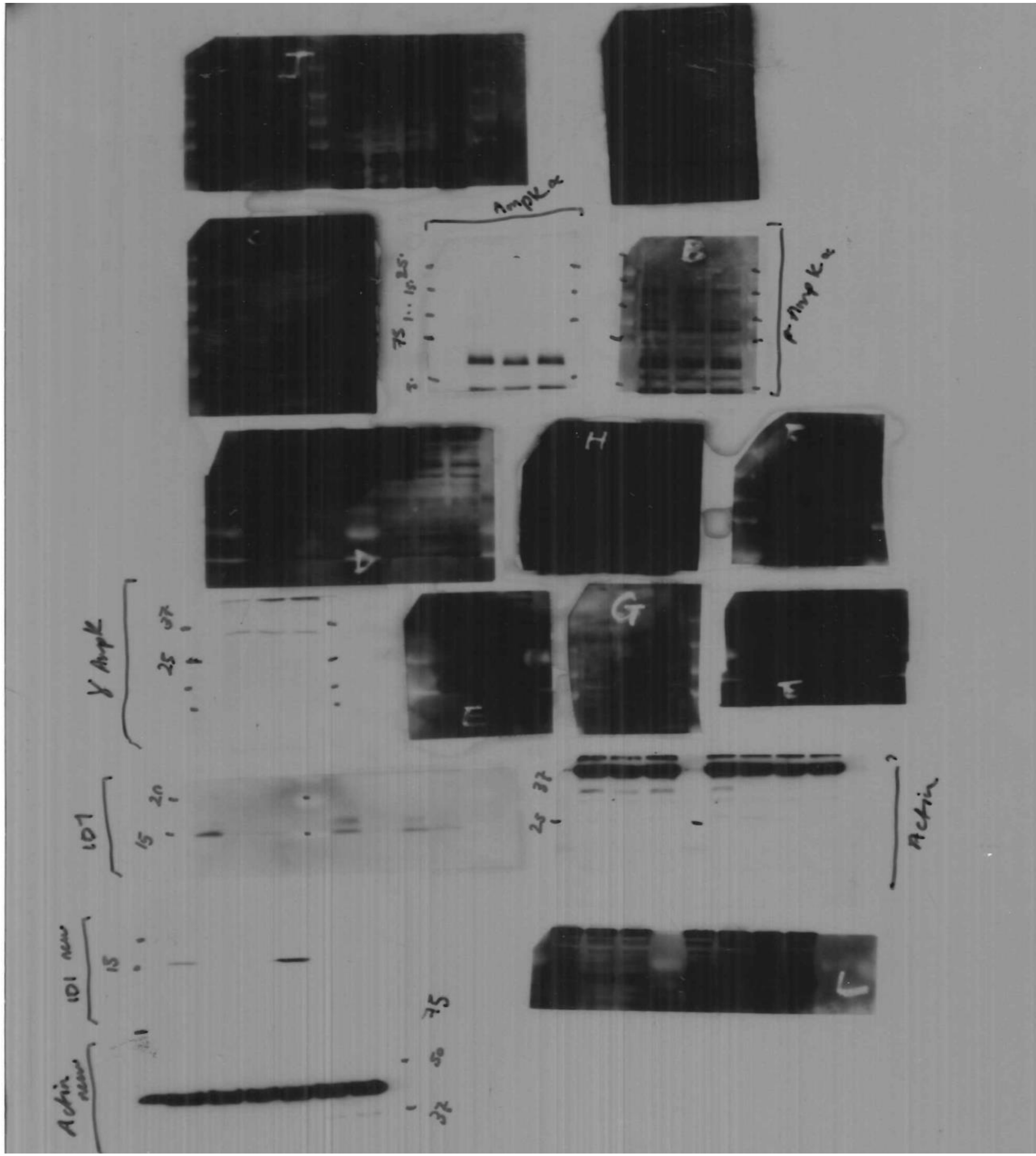

Supplementary figure F. Original western blot films pertaining to manuscript Figure 2-D.

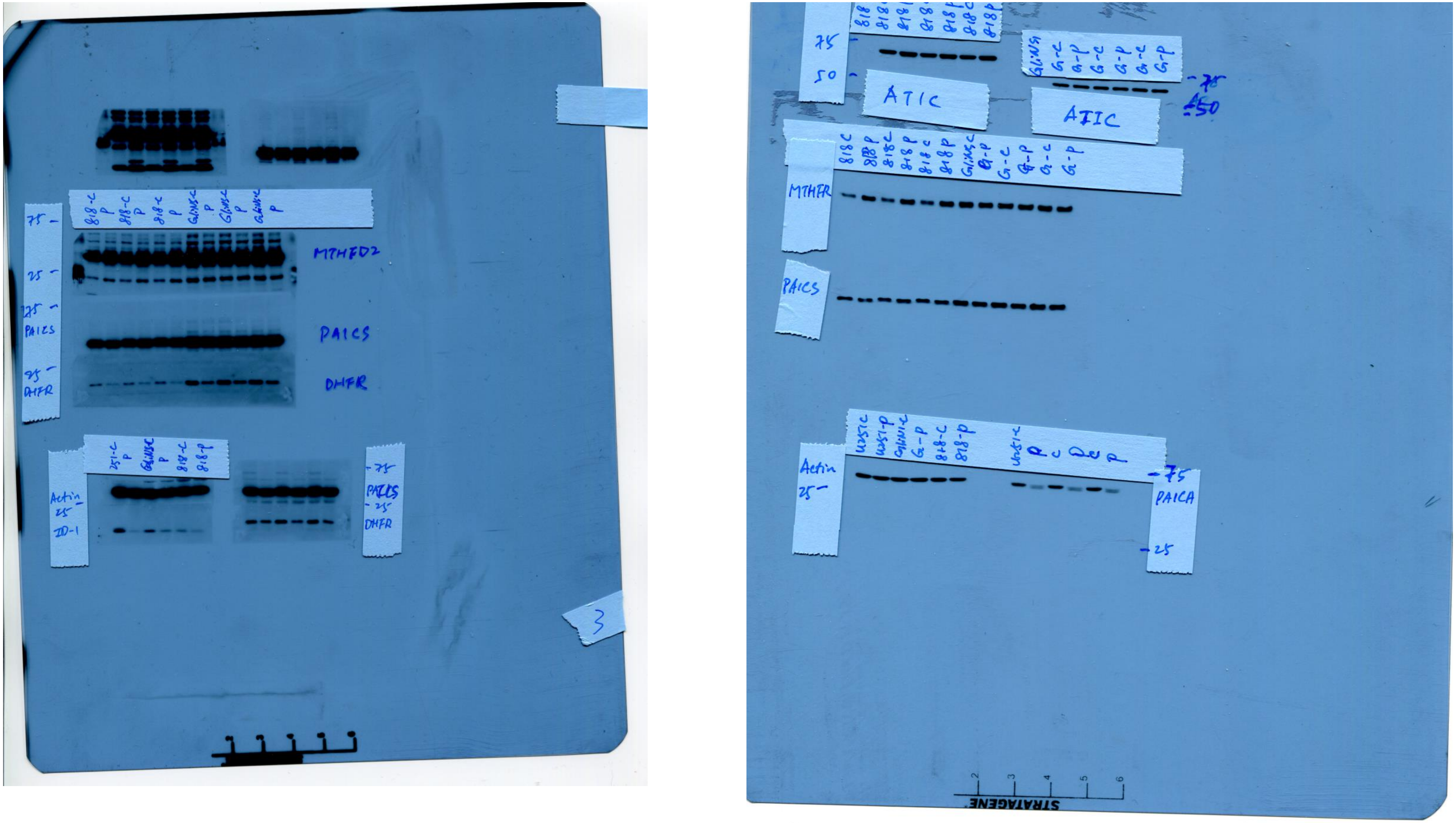

Supplementary figure G. Original western blot films pertaining to manuscript Figure 3

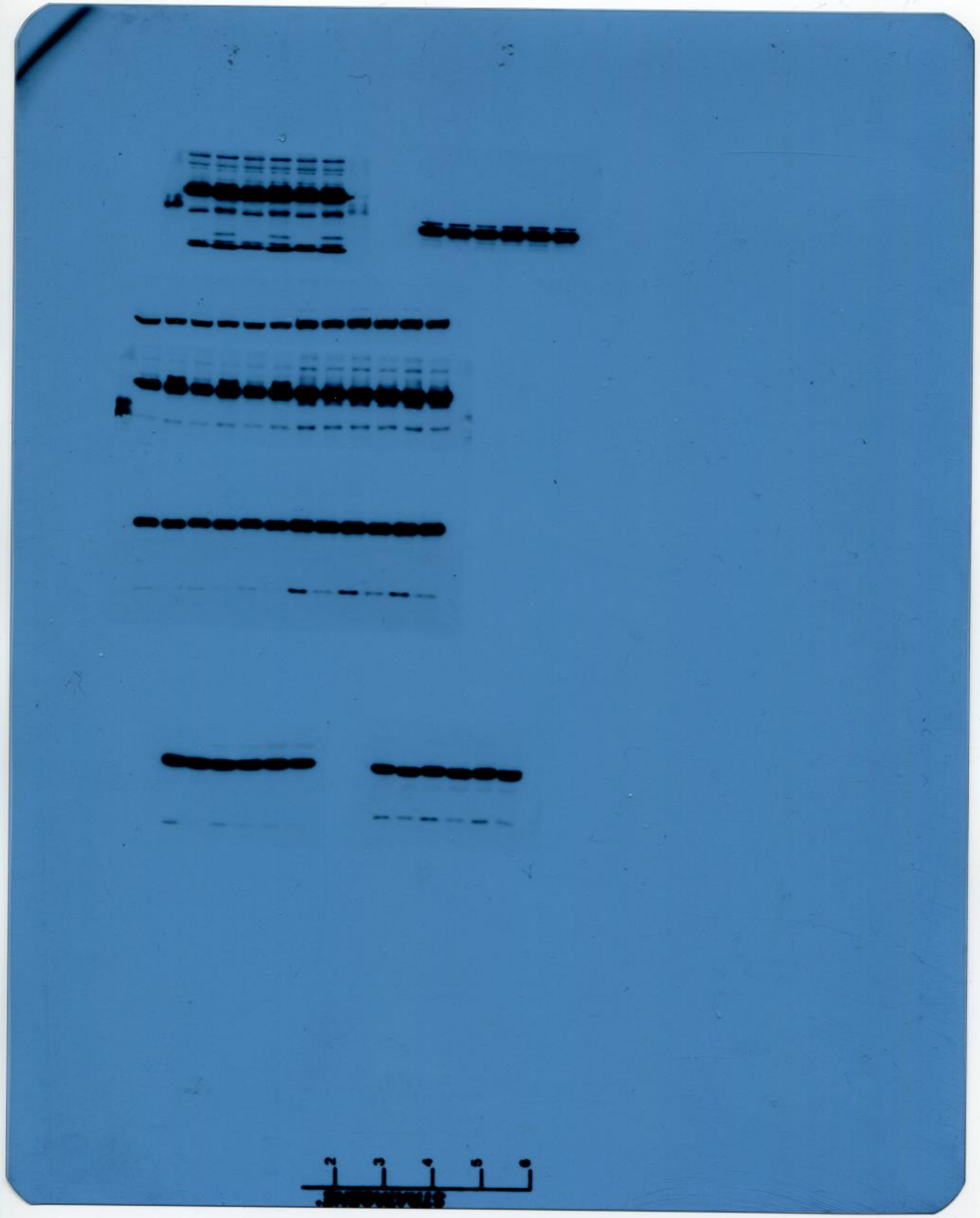

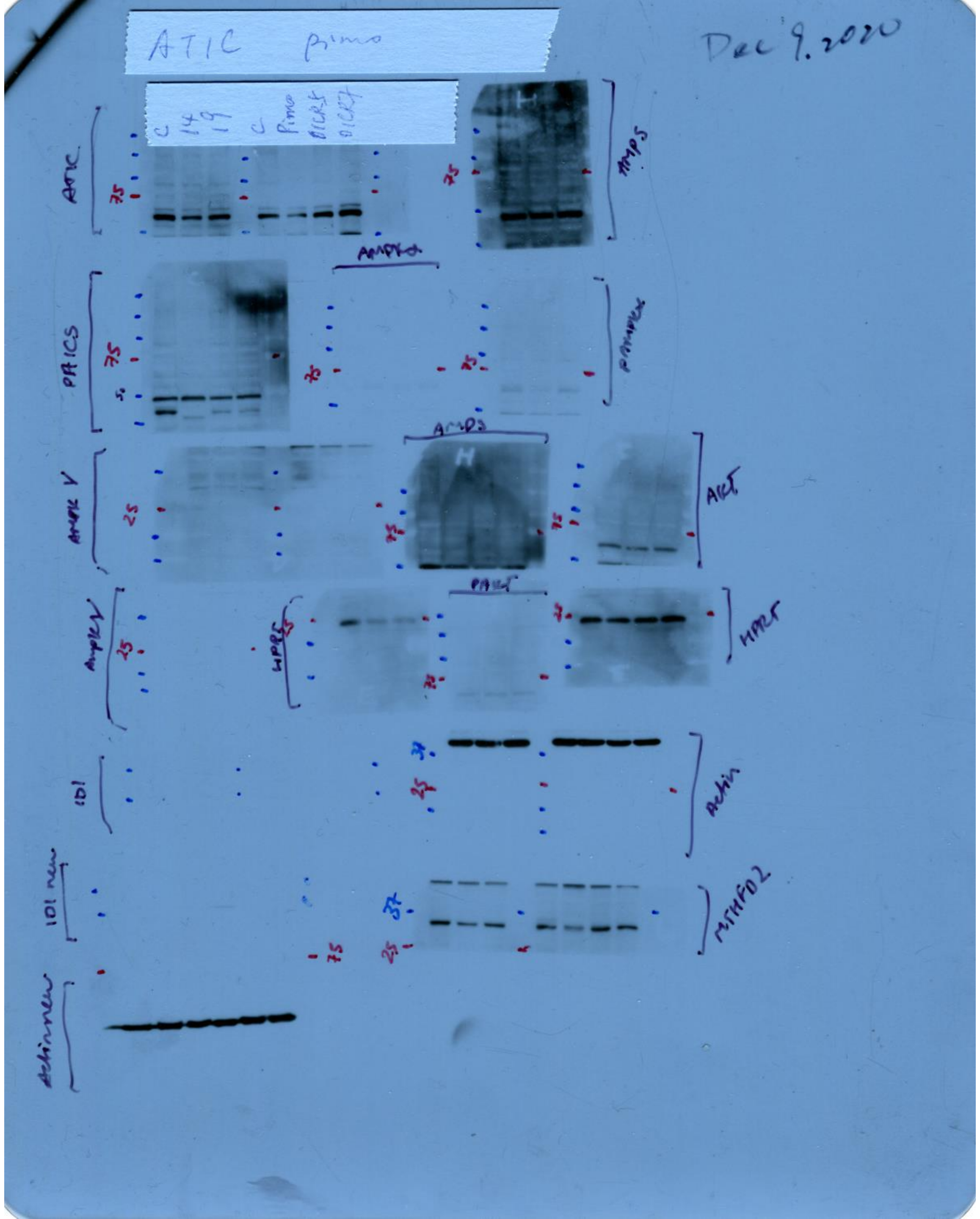

Supplementary figure H. Original western blot films pertaining to manuscript Figure 3.

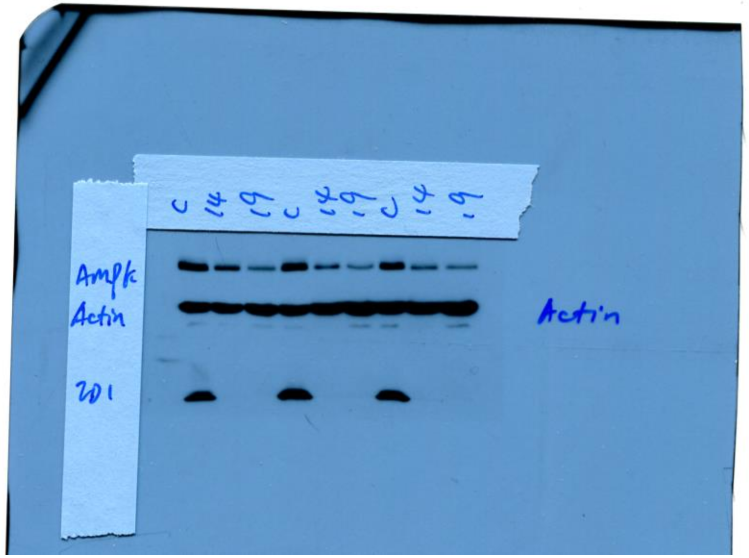

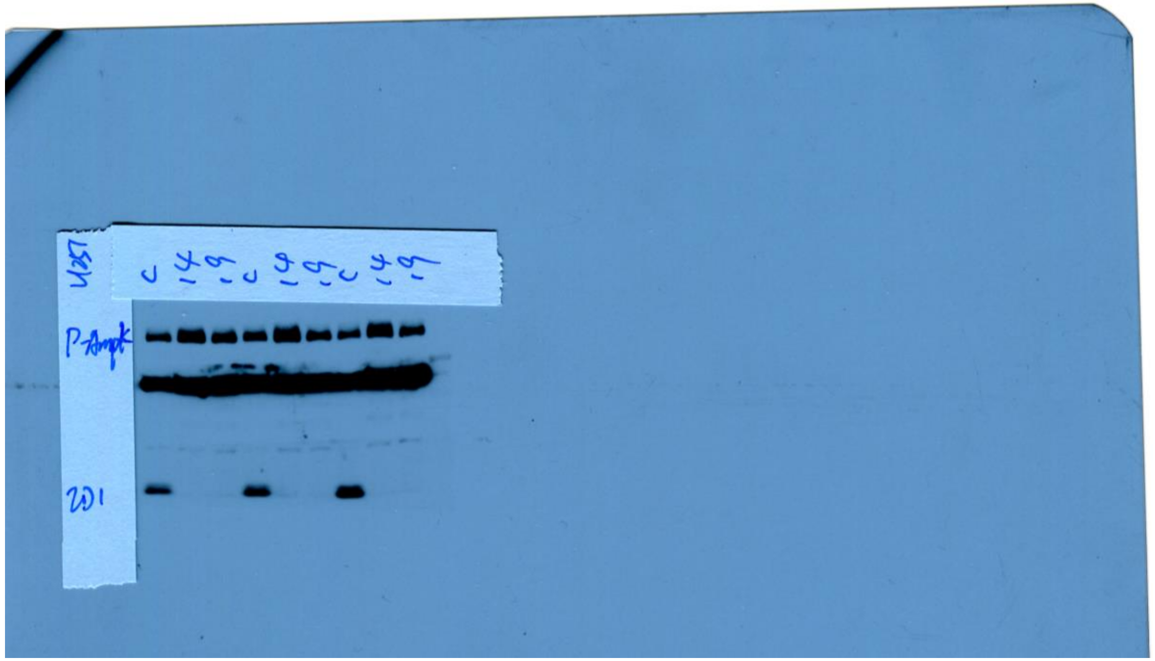

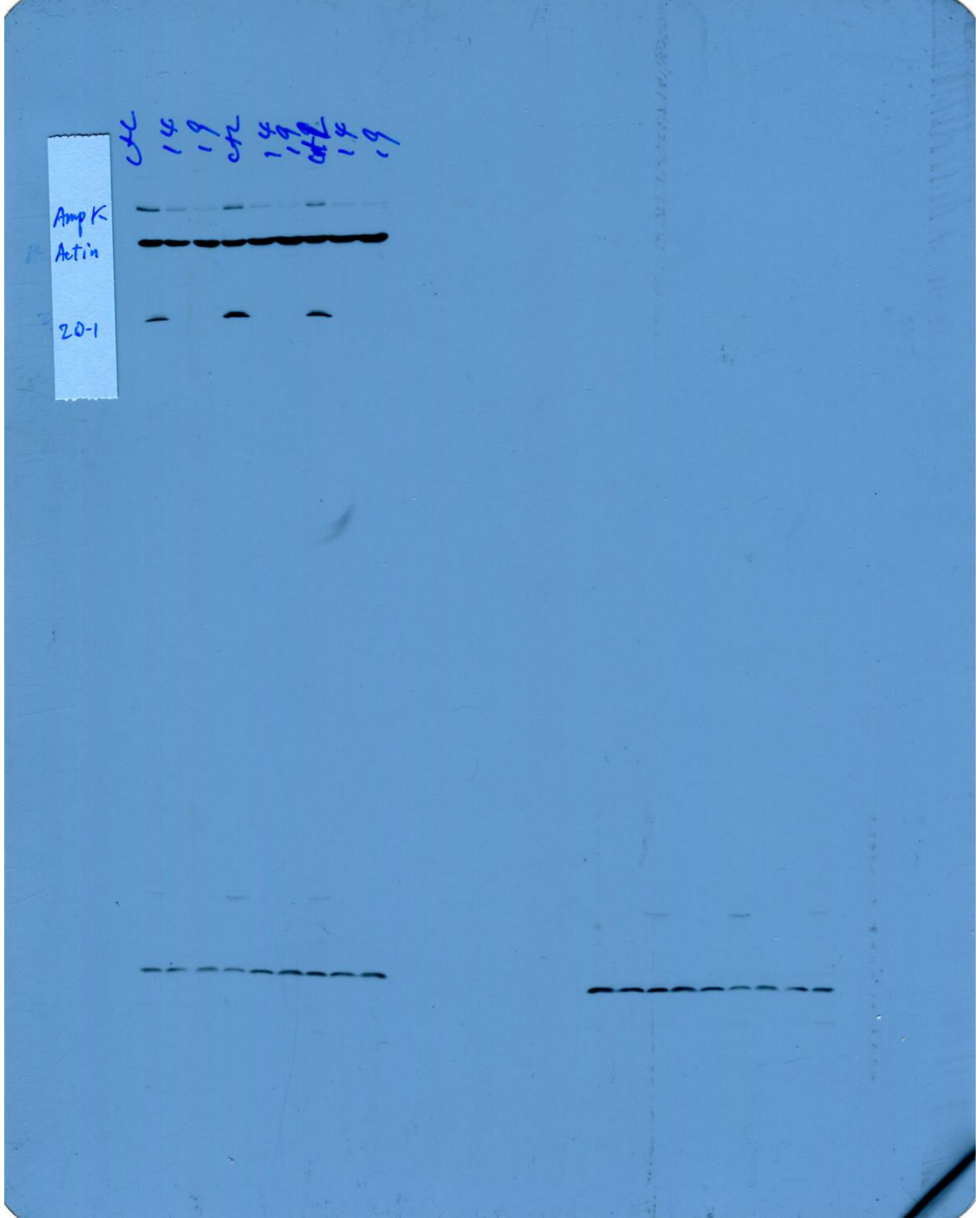

Supplementary figure I. Original western blot films pertaining to manuscript Figure 5.

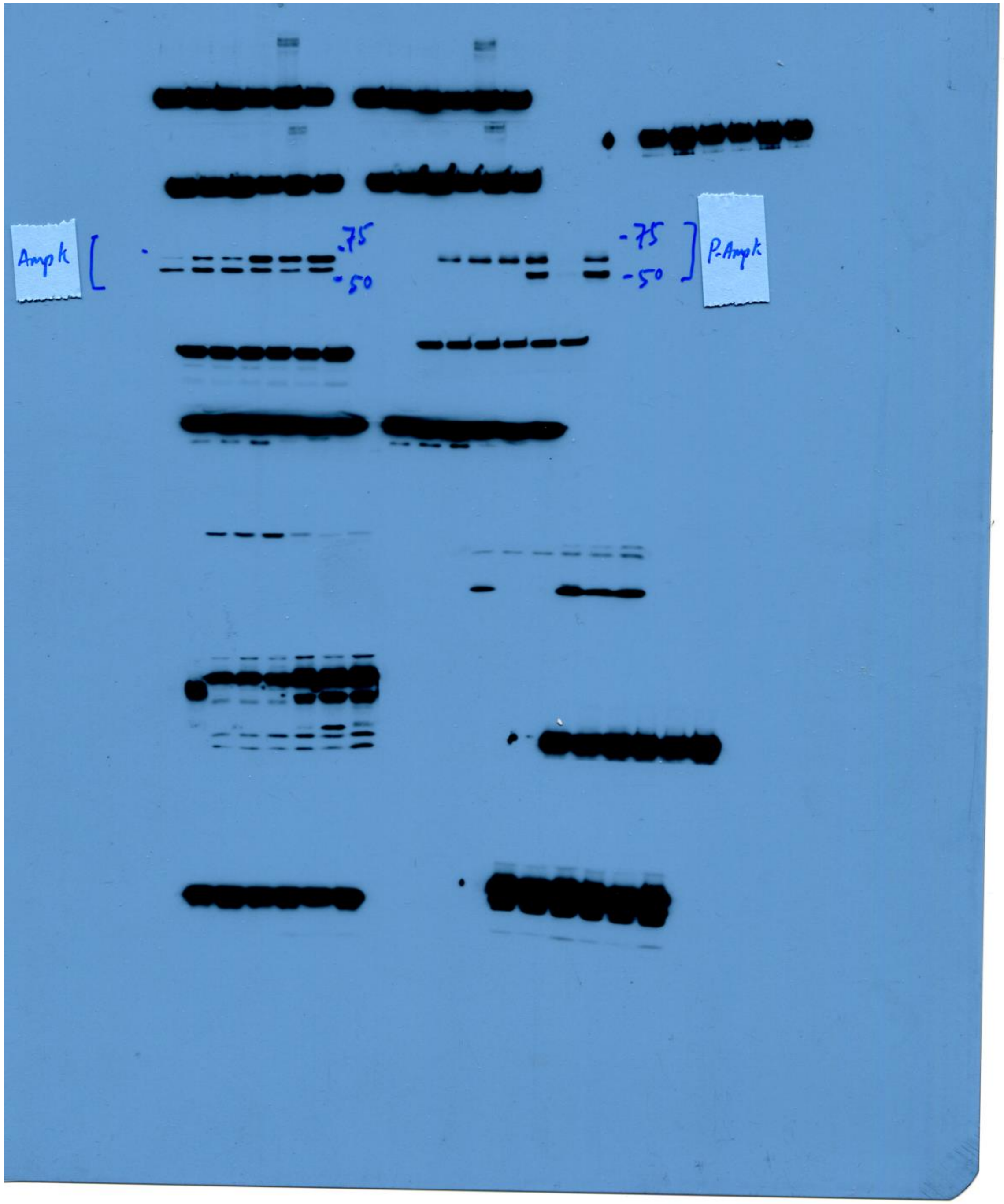

Supplementary figure J. Original western blot films pertaining to manuscript Figure 5.
